# A Scoping Review on Use of Drugs Targeting the JAK/STAT Pathway in Psoriasis

**DOI:** 10.3389/fmed.2022.754116

**Published:** 2022-02-25

**Authors:** Francisco Gómez-García, Pedro Jesús Gómez-Arias, Ana Montilla-López, Jorge Hernández-Parada, Juan Luís Sanz-Cabanillas, Juan Ruano, Esmeralda Parra-Peralbo

**Affiliations:** ^1^Inflammatory Immune-Mediated Chronic Skin Diseases' Laboratory, Insituto Maimónides de Investigación Biomédica de Córdoba (IMIBIC), Reina Sofia University Hospital, University of Cordoba, Córdoba, Spain; ^2^Department of Dermatology, Reina Sofia University Hospital, Córdoba, Spain; ^3^Department of Pharmacology, Reina Sofia University Hospital, Córdoba, Spain; ^4^Faculty of Biomedical and Health Sciences, Universidad Europea de Madrid, Madrid, Spain

**Keywords:** psoriasis, autoimmune diseases, JAK inhibitors, abrocitinib, deucravacitinib, ruxolitinib, tofacitinib

## Abstract

**Introduction:**

The Janus kinase–signal transducer and activator of transcription (JAK/STAT) pathway are known to be involved in inflammatory immune-mediated skin diseases, including psoriasis. The development of drugs targeting the JAK/STAT signaling pathway presents new treatment opportunities for psoriasis. However, the application of JAK inhibitors for the treatment of dermatological disorders is still in its early stages of development. This review summarizes available evidence in an attempt to identify knowledge gaps for conducting further research studies and improving clinical decision-making.

**Objective:**

The objective of this study is to conduct a scoping review of the use of drugs targeting the JAK/STAT pathway in the treatment of psoriasis.

**Methods:**

A priori protocol for scoping review was published in 2019. The Joanna Briggs Institute Reviewer's Manual and the PRISMA Extension for Scoping Review were used for the review. MEDLINE, EMBASE, CINAHL, Scopus, and Web of Science databases and ClinicalTrials registry were referred to in April 2019 and March 2021, respectively. References in English involving evidence on the use of drugs targeting the JAK/STAT pathway in patients with psoriasis were included. Data charting was performed by two authors using tables and figures.

**Results:**

The evidence found on the efficacy and safety of drugs targeting the JAK/STAT pathway in patients with psoriasis comes from 118 articles reporting the results of 34 randomized clinical trials (RCTs). Nine different drugs administered through various routes were identified (systemic: peficitinib, baricitinib, solcitinib, itacitinib, abrocitinib, deucravacitinib, and brepocitinib; topical: ruxolitinib; and both: tofacitinib). Knowledge articles are mainly created and published by pharmaceutical companies and authors through their own funding or by those related to them. Only tofacitinib and deucravacitinib have undergone phase III clinical trials, being the only ones tested with active comparators etanercept and apremilast, respectively. Proportions of Psoriasis Area and Severity Index (PASI) and Physician's Global Assessment (PGA) were the efficacy variables most frequently studied in systemic treatments. Only two RCTs declared the safety data collected by systematic assessment; the only systemic drug with phase III data was tofacitinib. Tofacitinib 5 mg two times daily (BID)/10 mg BID efficacy was compared with etanercept 50 mg/week and a placebo. At 12–16 weeks, PASI 75/PGA 01 ranges were as follows: 38.07–80%/37.16–67.4% for tofacitinib 5 mg BID; 54.79–100%/50–75.6% for tofacitinib 10 mg BID; 58.8/66.8% for etanercept, date from one only study; and 0–33.3%/9.04–33.3% for the placebo group. Other drugs in earlier stages of development showed values within these ranges. The most frequent adverse events (AEs) were nasopharyngitis and upper respiratory tract infections in all treatment groups.

**Conclusion:**

There is increasing evidence on the use of drugs targeting the JAK/STAT pathway as a treatment for psoriasis, although they are in the early phases of development. The trials conducted to date have been financed directly or indirectly by the pharmaceutical industry, which must be taken into account when interpreting the results of the trials. Psoriasis treatment is currently symptomatic and could potentially present a significant risk of toxicity. Therefore, the design of principal efficacy outcome measures considering the impact of the outcome on quality of life and a drug assessment methodology aimed at improving safety would probably strengthen the evidence and decision-making process.

## Highlights

- The use of drugs targeting the JAK/STAT pathway as a treatment for psoriasis is increasing, although they are in the early phases of development. Only tofacitinib and deucravacitinib have undergone phase III studies. None of the drugs have been approved yet.
- Most of the evidence produced so far is financed directly or indirectly by the pharmaceutical industry, which must be taken into account when interpreting the results.
- The most frequently used primary efficacy variables did not evaluate the quality of life. Few studies focus on safety, and most employ an unsystematic methodology. Standardized psoriasis-specific outcome measures would help reach better decisions.

## Introduction

Psoriasis is a chronic, immune-mediated dermatological disease with an estimated prevalence of 0.91–8.5% worldwide (1). Studies on quality of life in psoriasis patients demonstrate that disutility among psoriasis patients is within the same range as other chronic diseases, such as cancer, liver diseases, and diabetes (2). Associated comorbidities, such as cardiovascular risk, kidney disease, metabolic syndrome, or altered mood are related to a decrease in life expectancy (3). Finally, patients with psoriasis bear a higher financial burden due to absenteeism, in addition to the cost of managing their disease (4). Better knowledge of physiopathology has led to the development of molecules increasingly specific to the disease that reach high levels of efficacy. Despite this, the treatment of psoriasis remains symptomatic, and no treatment has been shown to address the basic cause of the disease and increase life expectancy in patients. In addition, they present a risk of potentially serious toxicity whereas high costs curtail the access of patients to these treatments and jeopardize the sustainability of health systems. Knowledge of all the available therapeutic alternatives allows cost-effective treatment recommendations to be adopted, which suit the values and preferences of patients.

From a pathogenic point of view, epidermal antigens activate dendritic cells resident in the dermis that converts naive T lymphocytes into functioning Th17 lymphocytes in a genetically permissive background (5). The presence of the HLA-C^*^06:02 risk allele, which codes an aminopeptidase that helps to process antigens for HLA class I presentation, and, specifically, the interaction with a risk variant in the ERAP1 gene, markedly increases the risk and therefore it implies to have a genetic background keen to psoriasis development for an individual (5). Interleukin 23 (IL-23) and Th-17 responses are considered important drivers of psoriasis, based on the findings from genome-wide association studies and clinical trials (5). Actually, psoriatic lesions result from hyperproliferation and disturbed differentiation of epidermal keratinocytes that are provoked by immune mediators of the IL-23 and IL-17 pathways (6). Th17 lymphocytes are believed to play a central role in the pathogenesis of psoriasis (7). In this context, the JAK/STAT pathway has been shown to participate in different key points of the pathophysiology of psoriasis, inducing the proliferation of Th17 lymphocytes (8) keratinocytes (9) and gamma–delta T cells. The regulation of these functions in the specified cell type is determined by the activation of the JAK/STAT pathway. The JAK/STAT pathway family is comprised of four types of cytoplasmic tyrosine kinases: JAK1, JAK2, JAK3, and Tyk2 (10), and seven transductors of the signal that activate translocation to the target gene expression: STAT1, STAT2, STAT3, STAT4, STAT5a, STAT 5b, and STAT6. STAT3 has recently emerged as a key player in the development and pathogenesis of psoriasis and psoriatic inflammatory conditions (7). JAK activation by IL-23 leads to the phosphorylation of STAT3 that transmits the signals of: IL-6, a key cytokine implicated in T17 cell programming; and also of IL-22, IL-19, IL-20, and IL-24 that act directly on keratinocytes (6). However, the complexity of the pathway is high, for example, although JAK 2 and TYK2 are fundamental for the transduction of the IL-23 signal, they are also involved in other pathways such as IL-10 or IL-13, which have protective roles in psoriasis (11). In this sense, polymorphins of TYK2 are known to protect against psoriasis (12).

In recent years, drugs acting on the JAK/STAT pathway have been developed by specifically inhibiting one component (filgotinib-JAK1, pacritinib-JAK2, and decernotinib-JAK3) or several of them (tofacitinib-JAK1 and JAK3; ruxolitinib, baricitinib-JAK1, and JAK2). These drugs have several advantages compared to biologics: they can be administered orally or topically and do not produce immunogenicity (7). Tofacitinib and upadacitinib, two JAK inhibitors, have been approved by both, Food and Drug Administration and European Medicine Agency (EMA), and only by EMA respectively, to treat psoriasic arthritis. However, none has been authorized for the use in skin psoriasis treatment.

A review of the scope is a mean for scientific synthesis that addresses an exploratory research question, with the objective of mapping key concepts and gaps in research related to a defined area or field (13).

In this work, we review the state of science on the study methodology used as well as the dissemination of the current knowledge on the drugs that block the JAK/STAT pathway in the treatment of psoriasis, what would allow to order it and detect gaps. This could be the base to formulate further specific research questions, which could be addressed by conducting a systematic review, later on (14).

The aim of this study is to present current evidence on the use of JAK inhibitors in the treatment of psoriasis, using a scoping review methodology.

## Materials and Methods

### Compliance With Ethics Guidelines

This article is based on previous studies and therefore does not include any study by any of the authors involving human participants or animals.

### Methods

A scoping review protocol has been published by us a priori (15). Our study was conducted and reported using the methodology described in the Joanna Briggs Institute Reviewer's Manual (16) and the PRISMA Extension for Scoping Reviews (17).

### Eligibility Criteria for Inclusion in Review

To be included in the review, papers had to show evidence of the use of JAK/STAT drugs in patients with psoriasis. Studies were included if they were written in English, involved human participants, and described the conditions formulated in the research question, regardless of the publication date or format. Articles were excluded if they did not fit the conceptual framework of the study. Non-scientific reviews were excluded from the analysis.

### Literature Search

Eligibility criteria and strategies for literature search are described in Supplementary Table 4.

### Data Charting

Two researchers jointly developed a data charting form to determine the variables to be extracted. A pilot test was conducted on five studies, and the chosen variables were included in a .csv file. The two researchers independently charted the data, discussed the results, and continuously updated the data charting form in an iterative process. Variables related to the study design and metadata from the primary sources are finally reported. Where possible, the data were collected from the clinical trial webpage; otherwise, data from congress abstracts and full-text articles were used.

### Collation, Summarization, and Reporting of Results

**Figure 1:**
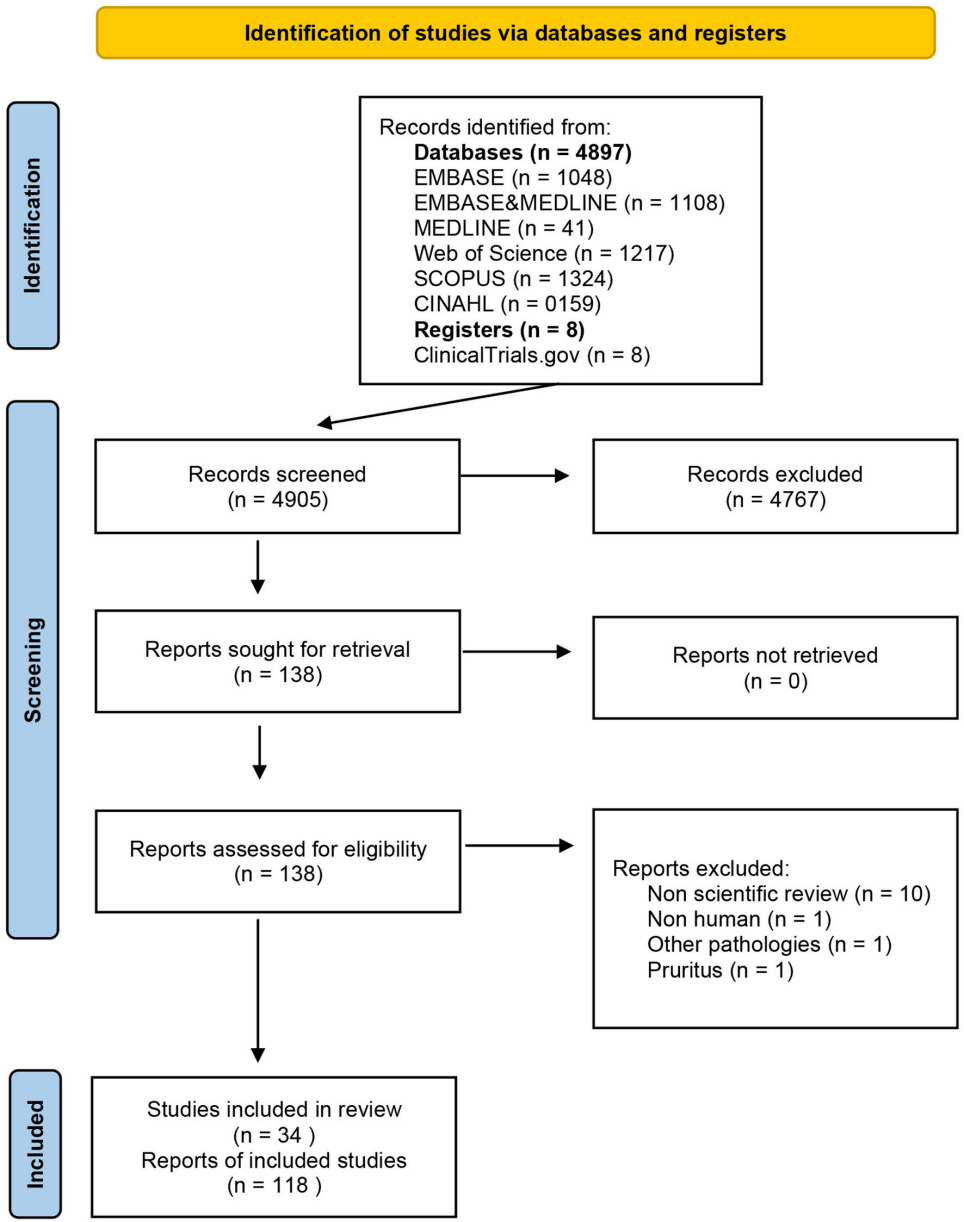
PRISMA diagram.

The results of the comprehensive research are presented using a PRISMA flow diagram (Figure 1). We first grouped the references and primary studies, drug-wise. Second, a narrative and qualitative synthesis of psoriasis mapping references, studies, and efficacy and safety data findings were elaborated using tables.

### Protocol vs. Scope Review

The review methods that are finally reported were compared with our planned search strategy published in *BMJ* (15). An update search was carried out using the ClinicalTrials registry in March 2021, for the anti-JAK-STAT drugs previously identified as used in the treatment of psoriasis.

## Results

### Search Results

**Figure 2:**
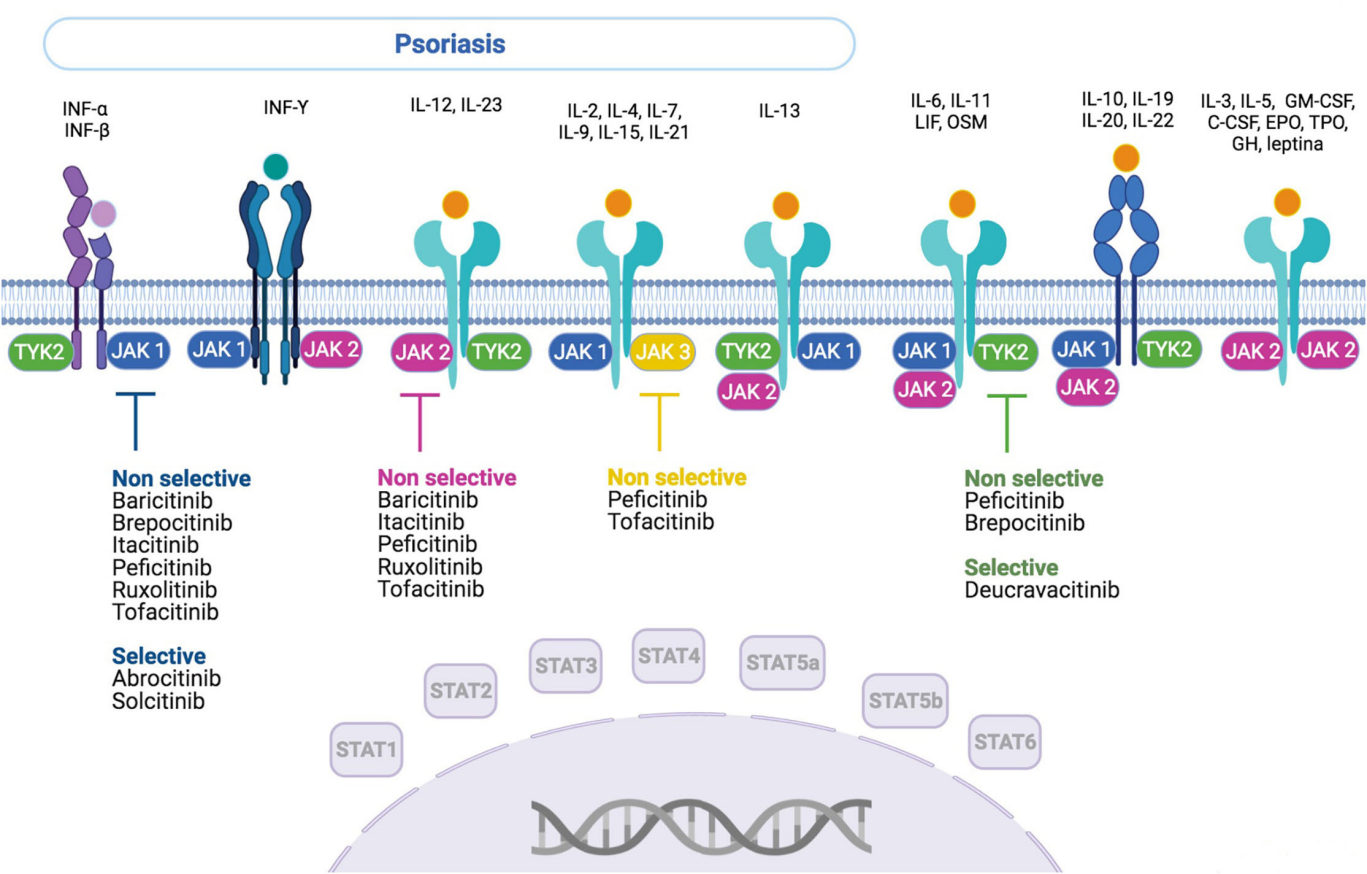
Anti-JAK drugs—action mechanism and selectivity. INF, interferon; IL, interleukin; OSM, oncostatin M; LIF, leukemia inhibitory factor; GM-CSF, granulocyte-macrophage colony-stimulating factor; C-CSF, granulocyte colony-stimulating factor; EPO, erythropoietin; TPO, thrombopoietin; GH, growth hormone.

From 4,897 records [EMBASE (*n* = 1.048), EMBASE and MEDLINE (*n* = 1,108), MEDLINE (*n* = 41), Web of Science (*n* = 1,217), SCOPUS (*n* = 1,324), and CINAHL (*n* = 159)] regarding the use of JAK/STAT-targeting drugs in the treatment of dermatological diseases, 130 references met the criteria for full-text review (Figure 1), after filtering out duplicates and selecting studies based on title, abstract, and keywords. Of these, 117 articles that belong to 26 different studies fulfilled the inclusion criteria. In March 2021, the list of previously identified anti-JAK drugs was updated with reference to the ClinicalTrials registry, adding one new reference and eight new studies. A total of 118 references and 34 studies (Supplementary Table 1) on nine drugs inhibiting the JAK/STAT pathway were found: tofacitinib, deucravacitanib, ruxolitinib, brepocitinib, peficitinib, baricitinib, solcitinib, itacitinib, and abrocitinib. These JAK inhibitors and their mechanisms of action and selectivity are shown in Figure 2. A reference list of all articles with reasons for inclusion and exclusion is presented in Supplementary Tables 5, 6.

Results pertaining to the nine drugs are listed below.

### Tofacitinib

#### Mapping References and Studies

A total of 103 references are shown in Supplementary Table 7: 93.2% (96/103), 4.8% (5/103), and 0.9% (1/103) of them correspond to studies on systemic, topical, and systemic topical tofacitinib treatment, respectively. Of these, 46.6% (48/103), 49.5% (51/103), and 3.8% (4/103) were full-text articles, congress abstracts, and letters, respectively. Most of them, that is, 80.5% (83/103), were published in dermatology journals. Overall, each publication was elaborated by 8.57 (1–17) authors: 4.76 (0–11), 1.31 (0–11), and 2.43 (0–9) author affiliations were to the pharmaceutical industry, research institutions, and dermatology departments of hospitals, respectively; A total of 56.3% (58/103) indicated collaboration among multinational centers, the US being the country whose centers contribute the greatest number of authors to the publications [75.8% (44/58)]. A total of 67.9% (70/103) and 66.0% (68/103) of the authors declared conflict of interests and funding sources, respectively. Among them, an average of 8.15 (0–17) authors declared a conflict of interest whereas 91.1% (62/68), 4.4% (3/68), and 4.4% (3/68) received funding from the pharmaceutical industry, public centers, and other sources, respectively. Pfizer Inc. [96.7% (60/62)] was the pharmaceutical company that funded the highest number of publications; 47.45% (28/59) of the publications, where the conflict of interest or type of funding was not declared, were congress abstracts.

Fifteen randomized studies—11 and 4 on systemic and topical treatments, respectively—were found (Supplementary Table 1). Studies on systemic treatment were conducted between November 2002 and June 2016. Of these, 10/11 (90.9%) and 6/11 (54.54%) were multinational studies and studies involving multiple centers, respectively. In seven studies, the US was the country with the highest number of participating centers. One phase-I study, two phase-II studies, and seven phase-III studies, with 59, 209, and 6,856 participants, respectively, of both sexes and older than 18 years, were funded by Pfizer. One study that included 18 patients was funded by the National Natural Science Foundation of China. The primary endpoints varied between 2 and 16 weeks. Three studies presented cohorts of 52 weeks. Maximum follow-up was undertaken at 67 months. Six doses of oral tofacitinib [2, 5, 10, 15, 20, 30, 50 mg, BID, and 60 mg once daily (QD)] were tested, with 5 mg BID and 10 mg BID being the most frequently investigated doses. The placebo and etanercept 50 mg administered subcutaneously two times a week were the only comparators evaluated. The primary objectives of the studies were efficacy (7/11), safety (2/11), efficacy or safety (1/11), and physiopathological aspects (1/11). The efficacy variables studied as primary objectives were PASI 75 and PGA 01 in four of the studies whereas mean reduction PASI was in one of the studies (Supplementary Table 2). Ten out of the 11 clinical trials declared that AEs were collected by non-systematic assessment.

Studies on the topical use of tofacitinib (Supplementary Table 1) were conducted between October 2008 and February 2015. Three out of the four studies were multinational studies involving multiple centers, most of which were located in the USA. One phase I and three phase-II studies, with a total of 15 and 618 participants, respectively, were funded by Pfizer. The primary endpoints were located between 12 days and 12 weeks. The latter was the period with the greatest long-term follow-up. Patients were 18 years of age or older, and both sexes were included. Tofacitinib 0.02, 0.2, 1, 2, and 4% were compared with the placebo and 50 μg/ml once or two times a day. The main objectives of the studies were related to efficacy variables. Two out of four clinical trials reported that AEs were collected by non-systematic assessment.

#### Tofacitinib Systemic Treatment

The efficacy variables PASI 75 and/or PGA 01 at 12–16 weeks of tofacitinib 5 mg BID, tofacitinib 10 mg BID, etanercept 50 mg/week, and the placebo were evaluated in eight (*n* = 1,221 patients), nine (*n* = 2,748 patients), one (*n* = 335 patients), and seven (*n* = 731 patients) studies, respectively (Supplementary Table 2). The values of PASI 75/PGA01 were in the range of 38.07% (*n* = 331)−80% (*n* = 5)/37.16% (*n* = 331)−67.4% (*n* = 43) for tofacitinib 5 mg BID; 54.79% (*n* = 2,200)−100% (*n* = 7)/50% (*n* = 8)−75.6% (*n* = 90) for tofacitinib 10 mg BID; 58.8% (*n* = 335)/66.8% (*n* = 335) for etanercept; and 0% (*n* = 6)−33.3% (*n* = 3)/9.04% (*n* = 177)−33.3% (*n* = 3) in the placebo group. Regarding security, most of the data were collected by non-systematic assessment (9/11), and the time frame was not specified (8/11). AEs were described for the different treatment arms at very short (14 days/one study), short (12–16 weeks/four studies), medium (24 weeks/one study), and long term (52 weeks/four studies, 66 months/one study), as shown in Supplementary Table 2. The most frequent AEs were nasopharyngitis and upper respiratory tract infections in all treatment groups. Severe AEs associated with tofacitinib are presented in Supplementary Table 8.

#### Tofacitinib—Topical Treatment

The efficacy of topical tofacitinib (Supplementary Table 9) at doses of 2% (*n* = 15) and 4% (*n* = 15) vs, placebo (*n* = 15) and calcipotriol 50 μg/g (*n* = 15) was evaluated at 12 days (improvement from baseline in psoriatic skin thickness/echo-poor band (EBP). Topical tofacitinib efficacy at four weeks resulted in an improvement in the Percent Change Target Plaque Severity Score (TPSS) at doses of 0.02% (*n* = 23), 0.2% (*n* = 23), and 2% (*n* = 71) vs. vehicle (*n* = 35). Finally, at 12 weeks, PGA improvement was observed in a study at a dose of 1% (*n* = 144) and 2% (*n* = 141) vs. the placebo (*n* = 145). At 12 days and 4 weeks, as cutoff primary points, no serious AEs, namely frequent burning or stinging, were observed. At 12 weeks, zero, seven, and four severe AEs were described in the tofacitinib 2%, 1%, and placebo groups, respectively.

### Ruxolitinib

Four references on topical ruxolitinib treatment—one full-text and three congress abstracts—were published between 2009 and 2012 (Supplementary Table 7). Overall, the studies were performed by a mean of eight authors (4–13), of which 6.25 (2–11), 1 (0–3), and 1.75 (0–3) had affiliations with the pharmaceutical industry, dermatology institutions, and other research institutions, respectively. Publications involved multiple centers, with three of the authors from the USA and only one from Spain. All the authors in one out of the four references—a full-text article (9)—declared conflict of interest whereas two out of the four references declared funding by the pharmaceutical group, Incyte Corp.

Three of the references mentioned above are experimental studies on topical treatment with ruxolitinib conducted between May 2007 and May 2009, two of which were randomized studies (Supplementary Table 1). All three studies were phase II clinical trials, with a total of 253 participants of both sexes ranging from 18 to 75 years in age. Three different doses of ruxolitinib cream (0.5, 1, and 1.5%) were tested against calcipotriene, betamethasone, and the placebo at cutoff points of 28 and 84 days. Two of these trials studied efficacy variables as primary outcome measures, and only one of them studied a safety variable. Only the results from one of the studies, NCT00820950, have been published; none of them have been posted in the clinical trial registry. All these studies were funded by the Incyte Corporation.

The efficacy and safety of topical ruxolitinib are shown in Supplementary Table 10.

### Peficitinib (ASP015K)

A full-text article and a congress abstract on systemic treatment using peficitinib were published in dermatology journals in 2012 and 2015, respectively (Supplementary Table 7). Studies were conducted by a mean of seven authors, four of them belonging to the pharmaceutical industry, and three of them to research centers. The publications involved multiple nations and centers, with the USA contributing the greatest number of authors. Only the full-text publication declared conflict of interests (all authors) and specified the funding source (Astellas).

A phase IIa randomized study on systemic treatment with peficitinib was conducted between March 2010 and July 2011 (Supplementary Table 1). It included 124 patients aged 18 years and over, of both sexes. Five oral doses of the drug—four, two times-daily dosing groups (10, 25, 60, and 100 mg) and one once-daily dosing group (50 mg)—were compared with the placebo at 6 weeks. Efficacy, reduction of PASI 75, and safety variables were among the primary outcome measures studied. We did not find a description of the safety outcomes in the publications or on the clinical trial webpage. This study was funded by Astellas.

The efficacy and safety at 6 weeks are summarized in Supplementary Tables 3, 11.

### Baricitinib

Four references on systemic treatment using baricitinib were found, three of which were published in dermatology journals and one in a general medicine journal between 2014 and 2019 (Supplementary Table 7). Three of them were full-text articles, and the other was a congress abstract. Studies were conducted by a mean of 7.5 (6–9) authors, of which 5.5 (3–9) had affiliations to the pharmaceutical industry. All involved multiple centers, and three were multinational, with the USA contributing the greatest number of authors. Conflict of interests (all the authors) and funding by the pharmaceutical industry (all funded by Eli Lilly) were declared in all three full-text references.

A randomized phase IIb study of systemic treatment with baricitinib was conducted between December 2010 and August 2014 (Supplementary Table 1). A total of 271 patients of both sexes, 18 years of age or older, were included. Four oral doses of baricitinib (2, 4, 8, and 10 mg) were compared with the placebo after 12 weeks of treatment. One primary outcome measure of efficacy, the PASI 75, was assessed. AEs were collected by systematic assessment.

The study was funded by Eli Lilly.

The efficacy and safety results at 12 weeks are presented in Supplementary Tables 3, 12. Serious baricitinib AEs are summarized in Supplementary Table 13.

### Solcitinib

A full-text reference on systemic treatment using solcitinib was published in a dermatological journal in 2016 (Supplementary Table 7). The publication was multinational involving multiple centers, with the USA contributing the greatest number of authors. A total of 12 authors, 10 of whom had a pharmaceutical industry affiliation and two of whom had a dermatology center affiliation, contributed to this study. The authors declared that conflict of interests were involved. The study was funded by GlaxoSmithKline.

A randomized phase-IIb study on systemic treatment with solcitinib was conducted from March 2013 to March 2014 (Supplementary Table 1). A total of 68 patients aged 18–75 years, of both sexes, were included. Three oral doses of solcitinib (100, 200, and 400 mg) were compared with the placebo after 12 weeks of treatment. PASI 75 was assessed as the primary outcome measure of efficacy. AEs were collected through systematic assessment. This study was funded by GlaxosmithKline.

The efficacy and safety results at 12 weeks are summarized in Supplementary Tables 3, 14. Serious solcitinib AEs are shown in Supplementary Table 15.

### Itacitinib

A full-text reference on systemic treatment with itacitinib was published in a dermatological journal in 2016 (Supplementary Table 7). The publication was multinational involving multiple centers, with the USA contributing the greatest number of authors. A total of 11 authors (nine from the pharmaceutical industry and two from research institutions) contributed to this study, nine of whom declared a conflict of interest. It was funded by the Incyte Corporation.

A phase II study on systemic treatment with itacitinib was conducted between June 2012 and February 2013 (Supplementary Table 1). A total of 50 patients of both sexes, aged 18–75 years, were included in the study. Four oral doses (100 mg QD, 200 mg QD, 200 mg BID, and 600 mg QD) were compared with the placebo at 28 days. The efficacy, PGA change, and primary safety objectives were evaluated. We did not find a methodology for AE assessment in the publications or on the clinical trial webpage. The study was funded by the Incyte Corporation.

The results for efficacy and safety after 28 days of treatment are presented in Supplementary Tables 3, 16, 17.

### Deucravacitinib (BMS-986165)

A full-text reference on (BMS-986165) systemic treatment with deucravacitinib was published in a general medicine journal in 2018 (Supplementary Table 7). The study was multinational involving multiple centers, with the USA contributing the greatest number of authors. The study was conducted by nine authors (three from the pharmaceutical industry, two from dermatological institutions, and four from other research institutions). The authors declare that conflict of interests were involved. The study was funded by Bristol Myers Squibb.

Eight studies, one in phase I, one in phase-II, and six in phase III with 140, 268, and 3,690 patients, respectively, of both sexes and all ages on systemic deucravacitinib treatment, were conducted from November 2016 to April 2024 (Supplementary Table 1). Six of these eight clinical trials studied the primary efficacy variables, PASI and PGA. Three oral doses (3 mg QD, 3 mg BID, and 6 mg BID) were compared to the placebo, apremilast, famotidine, and interferon 2alfa recombinant at 12 or 16 weeks. We did not find an AE assessment methodology. This study was funded by Bristol Myers Squibb.

The efficacy and safety at 12 weeks are summarized in Supplementary Tables 3, 18.

### Abrocitinib (PF-04965842)

A full-text reference on the systemic treatment with abrocitinib was published in a dermatology journal in 2018 (Supplementary Table 7). The publication was uninational (USA), involving multiple centers. A total of 12 authors (nine, one, and two from the pharmaceutical industry, a dermatological institution, and a research institution, respectively) contributed to this study. The authors declare no conflict of interest. This study was funded by Pfizer.

A phase-II study on systemic treatment with abrocitinib was conducted between November 2014 and September 2015 (Supplementary Table 1). A total of 59 patients of both sexes, aged 18–65 years, were included. Three oral doses (200 mg QD, 400 mg QD, and 200 mg BID) were compared with the placebo at 4 weeks. The PASI was evaluated as a primary objective. AE was collected by a non-systematic assessment. This study was funded by Pfizer.

The efficacy and safety results are presented in Supplementary Tables 3, 19.

### Brepocitinib (PF-06700841)

A full-text reference on systemic treatment with brepocitinib was published in a pharmacology journal in 2017 (Supplementary Table 7). The publication (USA) involved multiple centers. A total of 11 authors (10 from the uninational pharmaceutical industry and one from a research institution) contributed to this study. All authors declare that they have no conflict of interest. This study was funded by Pfizer.

Three studies, one in phase-I and two in phase-II, on systemic treatment with brepocitinib, with 96 and 452 patients, respectively, of both sexes ranging from 18 to 75 years in age, were conducted from November 2014 to April 2021 (Supplementary Table 1). Seven oral doses, ranging from 30 mg QD to 100 mg QD, were compared to the placebo at four and 12 weeks. As primary objectives, PASI 75 was evaluated as a primary objective in two of these studies whereas pharmacokinetics and arterial pressure in the other one. The primary objectives namely safety, pharmacokinetics, efficacy, and PASI reduction were evaluated. AE was collected by a non-systematic assessment. These studies were funded by Pfizer.

No efficacy data were found. Safety data are presented in Supplementary Tables 3, 20.

## Discussion

### Summary of Findings

To our knowledge, this is the first scoping review on the use of drugs targeting the JAK/STAT pathway for treating psoriasis. Nine molecules that inhibit the JAK/STAT pathway were identified. Some of these drugs act on a single-specific component of this pathway, such as abrocitinib and solcitinib (JAK1) and deucravacitinib (TYK2), whereas others do so by inhibiting several components, such as baricitinib, ruxolitinib, itacitinib (JAK1 and JAK2), brepocitinib (JAK1 and TYK2), tofacitinib (JAK2 and JAK3), and peficitinib (JAK1, JAK2, JAK3, and TYK2). All of them, except ruxolitinib applied topically, have been used orally. Tofacitinib was the only drug tested in both forms of administration. These drugs are in different stages of development. Most drugs are being tested in phase-II studies; only tofacitinib and deucravacitinib are being tested in phase-III studies. None of these drugs have been approved for use in the treatment of psoriasis.

The evidence available so far comes mainly from clinical trials that are promoted almost entirely by the pharmaceutical industry which also funds the notification of the results and conclusions from those studies. The dissemination of knowledge is mainly carried out through journals and congresses related to dermatology by authors belonging to the pharmaceutical industry with declared conflict of interests. Results from some of the registered studies have not been published after the completion of the trials. All systemic treatments have been compared mainly to the placebo, tofacitinib, and brepocitinib being the only drugs that have been tested against other active molecules, specifically, against etanercept and apremilast, and against famotidine, and interferon 2 alpha recombinant, respectively. Drugs administered topically include the placebo, calcipotriol, and betamethasone. The primary objectives of these clinical trials focus mainly on aspects of efficacy rather than safety and present primary endpoints in the short (12–16 weeks) or very short term (days−4 weeks). The effectiveness, measured as the reduction in PASI, PASI 75, and PGA, varies depending on the tested dose. Most of the data regarding security were collected by non-systematic assessments. The short-term data were similar between the different treatment arms, with nasopharyngitis being the most frequent AE. Tofacitinib was the only drug with long-term data available.

### Strengths and Limitations of the Review

Regarding the methodology of this study, the study was conducted based on an a priori protocol previously published in a scientific journal and using the latest standards in scoping review methodology; at least two researchers were involved in each phase. This manuscript was prepared according to the recommendations of the PRISMA Extension for Scoping Reviews. We also identified a high number of anti-JAK drugs whose current development phase made them eligible for inclusion in the latest Cochrane living review update (18).

Limitations related to funding and time prevented us from including articles written in languages other than English. Additionally, we were unable to contact the authors of some articles that would have helped reduce the amount of missing data, particularly for studies published as congress abstracts, as we did not exclude these types of publications. This work is a substudy, and although we believe that the global search strategy was a complete one, and that the three-phase search minimized overlooking of relevant articles, it is still possible that we did not include some articles describing studies related to the research topic. In March 2021, an update of the previously identified anti-JAK drugs was carried out, but only on the clinical trial webpage. Finally, most of the studies have been carried out, founded, and disseminated by pharmaceutical industry, and the validity of the conclusions may be comprised.

### Findings in Context and Research Gap

The creation and notification of knowledge about drugs that act on the JAK/STAT pathway are funded almost exclusively by the pharmaceutical industry. Further, knowledge diffusion is carried out by authors with conflict of interest, most of whom belong to the pharmaceutical industry. In addition, a high percentage of references are congress abstracts that are not subjected to any peer review process, and it is a known fact that the products of sponsors are favored (19). Also, it is common knowledge that between two-thirds and three-quarters of randomized trials reported in major journals have been supported by the pharmaceutical industry (20). There is strong evidence to show that compared to independent trials, industry-funded studies exaggerate treatment effects in favor of products promoted by their sponsor (21). Furthermore, industry-sponsored trials are more likely than other trials to conclude that a drug is safe (22). Thus, independent studies are necessary. Alternatively, external evaluators could access the primary studies and participate in the dissemination of the results. A meta-epidemiological study has found that randomized clinical trials using routinely collected data to assess outcomes indicate systematically less favorable treatment effects than trials using traditional methods used in the clinical trials considered in this review. In this context, using data from clinical patient registers, mobile devices, or electronic health records may improve the validity of the results of treatments (23). Further, we found clinical trials whose results have not been published or have not been included in clinicalTrials.org; there is evidence of a delay of more than 7 years in the publication of the results after the study completion of up to 25% of them (24). There is evidence on how selective reporting of studies poses a risk to the health of patients (25). All the above factors must be considered when evaluating the knowledge available on these drugs at the time of evaluation.

The objective of the studies is found to most frequently focus on the efficacy outcomes, whose readout is the extension of the lesions, PASI, and PGA. Although these outcomes are the most widely used in the trials of drugs for psoriasis, standard measurement criteria are essential for the results to be accepted by the clinical community. However, it is also true that a key determining factor of the scientific value of clinical trials is the choice of measures of outcomes (26). In this sense, bestowing more importance on the influence of the surface body in reducing the quality of life is questionable; this impact is influenced by factors depending on the location of lesions (palms, plants, and visible and stetic-disfigured regions). In fact, symptoms of pain or itching, the presence of comorbidities, and being older or female are the factors that are most clearly associated with a decrease in quality of life (27). Therefore, it is possible that the efficacy measured in these trials was not the most useful for clinical extrapolation in patients. Here, the Cochrane Skin Core Outcome Set Initiative is of great interest, as it has been recently established to improve and standardize outcome measurement in clinical trials and to make the evidence more useful (28). Regarding safety, the facts that most of the data were collected by non-systematic assessments and that the time frames were not specified make it difficult to interpret the findings. In this sense, a better methodology for collecting and reporting results is desirable. In addition, knowledge of safety is focused on the short or very short term, making the uncertainties high, necessitating better collection and notification of new data from more studies.

## Conclusion

The number of drugs targeting the JAK/STAT pathway for treating psoriasis is increasing, tofacitinib being the most widely known. The evidence available must be interpreted considering that the funding for conducting studies on these drugs and notification of their results comes mainly from the pharmaceutical industry. The sources of knowledge are RCTs, whose primary objectives are focused on the issues of efficacy rather than safety, and their cutoff points are located in the very short or short term; we put evidence enough together to point out that principal efficacy primary outcome scales did not take into account fundamental aspects that impact the quality of life, such as symptoms and the location of the lesions, which are very variable depending on the doses administered. Also, only tofacitinib and deucravacitinib are being tested in phase III clinical trials. The methodology used in investigating and reporting on the safety of the drugs used suggests that the current high level of confidence in the findings of these studies is overrated.

## Data Availability Statement

The original contributions presented in the study are included in the article/Supplementary Material, further inquiries can be directed to the corresponding author.

## Author Contributions

JR and FG-G: conceptualization. FG-G and EP-P: data curation and writing—original draft. JR: funding acquisition. FG-G, PG-A, AM-L, JH-P, JS-C, and EP-P: investigation. JR and EP-P: supervision. FG-G, EP-P, and PG-A: validation. JR, FG-G, and EP-P: visualization. EP-P: writing—review and editing. All authors contributed to the article and approved the submitted version.

## Funding

This work was supported, in part, by project ICI1400136 to JR, integrated into the National Plan of R+D+I 2008-2011, and cofinanced by the ISCIII-Subdirección General de Evaluación and European Regional Development Fund (ERDF), project PIN-0316-2017 of the Consejería de Salud, Junta de Andalucía (Spain) to JR, and by Grant PP13/009 of Plan Propio de movilidad para investigadores del Instituto Maimonides de Investigacion Biomédica de Córdoba (IMIBIC). The funders had no role in the study design, data collection and analysis, decision to publish, or preparation of the manuscript.

## Conflict of Interest

FG-G has received honoraria for research from Pfizer and for lecturing from AbbVie, Janssen-Cilag, and Novartis. JR has received honoraria for lecturing and grants for research from Pfizer, honoraria for lecturing from Janssen-Cilag and Novartis, and other financial benefits from AbbVie and Novartis. The remaining authors declare that the research was conducted in the absence of any commercial or financial relationships that could be construed as a potential conflict of interest.

## Publisher's Note

All claims expressed in this article are solely those of the authors and do not necessarily represent those of their affiliated organizations, or those of the publisher, the editors and the reviewers. Any product that may be evaluated in this article, or claim that may be made by its manufacturer, is not guaranteed or endorsed by the publisher.
