# Supplementary material for: A Scoping Review on Use of Drugs Targeting the JAK/STAT Pathway in Psoriasis
Source: Front Med (Lausanne). 2022 Feb 25;9:754116. doi: 10.3389/fmed.2022.754116 (PMC8914468; doi:10.3389/fmed.2022.754116)
Supplement: Supplementary file 1 [file Data_Sheet_1.docx]

**Table S1. Mapping studies**

Abbreviations: BID, *'bis in die'*, twice a day. QD, '*quaque die*', every day.

| **Drug / Topical-systemic/**  **Clinical trials or study number** | **N (patient enrollment)**  **/study-phase/start- completion date** | **Number**  **of references** | **Age-sex**  **Participants/**  **End points** | **Intervention** | **Number endpoints / Type Primary**  **Endpoints / Specific primary outcomes** | **Sponsor**  **Locations**  **Countries**  **Principal country** |
| --- | --- | --- | --- | --- | --- | --- |
| Tofacitinib  Systemic  NCT01736696 | N=59  1  11-2002-  04-2004 | 1 | 18-65 years old  All  59  14 days | Tofacitinib 5 mg, 10 mg, 20 mg, 30 mg, 50 mg BID and 60 mg QD | 42/safety/ Change from baseline in QT interval | Pfizer  1  1  EEUU |
| Tofacitinib  Systemic  NCT00678210 | N=197  2  07-2008  08-2009 | 20 | 197  12 weeks | Tofacitinib 2mg **BID**, 5mg **BID**, 10mg **BID**/Placebo | 1/efficacy/  PASI 75 | Pfizer  44  2  EEUU |
| Tofacitinib  Systemic  NCT01710046 | N=12  2a  03-2013  11-2013 | 1 | 18 years and older  All  12  12 weeks | Tofacitinib 10 mg BID  Placebo | 2/efficacy/  PASI 75/ PGA 0-1 | Pfizer  5  1  EEUU |
| Tofacitinib  Systemic  NCT01186744 | N=666  3  09-2010  01-2013 | 12 | 18 years and older  All  666  4-56 weeks | Tofacitinib  5mg **BID**, 10 mg BID/**Placebo** | 4/efficacy/  PASI 75/ PGA | Pfizer  87  12  EEUU |
| Tofacitinib  Systemic  NCT01241591 | N=1101  3  **11-**2010-  01-2013 | 15 | 18 years and older  All  1101  12 weeks | Tofacitinib 5mg **BID**, 10 mg BID  Etanercept 50 mg twice weeks  Placebo | 2/efficacy/  PGA 0-1/ PASI 75 | Pfizer  122  26  Germany |
| Tofacitinib  Systemic  NCT01309737 | N=960  3  03-2011  04-2013 | 22 | 18 years and older  All  960  16-52weeks | Tofacitinib  5 mg **BID**,  10 mg BID  Placebo | 2/efficacy/  PGA 0-1 / PASI 75 | Pfizer  94  11  EEUU |
| Tofacitinib  Systemic  NCT01276639 | N=901  3  03-2011  04-2013 | 23 | 18 years and older  All  901  16-52 weeks | Tofacitinib 5 mg. **BID**,10 mg BID  Placebo | 2/efficacy/  PGA 0-1 / PASI 75 | Pfizer  74  11  EEUU |
| Tofacitinib  Systemic  NCT01163253 | N=2867  3  09-2010  06-2016 | 7 | 18 years and older  All  2867  4 weeks-67 moths | Tofacitinib 5 mg **BID**, 10 mg BID | 55/ safety/ Emergent Adverse Events (AEs) and Serious Adverse Events (SAEs)/ clinical and analytical parameters changes | Pfizer  323  33  EEUU |
| Tofacitinib  Systemic  NCT01519089 | N=95  3  03-2012  01-2014 | 1 | 20 years and older  All  95  16-52 weeks | Tofacitinib 5 mg **BID,** 10 mg BID | 5/efficacy-safety  PASI 75/ PGA 0-1 | Pfizer  16  1  Japan |
| Tofacitinib  Systemic  NCT01815424 | N=266  3  12-2013  07-2015 | 1 | 18 years and older  All  266  16 weeks | Tofacitinib 5 mg **BID**, 10 mg BID  Placebo | 2/efficacy  PGA 0-1/ PASI 75 | Pfizer  24  3  China |
| Tofacitinib  Systemic | Serum Human Beta-Defensin-2 Is a Possible Biomarker for Monitoring Response to JAK Inhibitor in Psoriasis Patients  NA/NA | 1 | 37-59 years  All  18  8,16 weeks | Tofacitinib 5mg, BID,  Tofacitinib 10 mg BID | Efficacy/  PASI 75 | National Natural Science Foundation of China  2  1  China |
| Tofacitinib  Topical  NCT02193815 | N=15  1  09-2014  02-2015 | 0 | 18 years and older  All  15  12 days | Tofacitinib 4% daily solution, 2% daily ointment, vehicle, Calcipotriol | 1/efficacy/ Change From Baseline in Psoriatic Skin Thickness/Echo-Poor Band (EPB) | Pfizer  1  1  Germany |
| Tofacitinib  Topical  NCT01246583 | N=71  2  02-2011  09-2011 | 3 | 18-65 year old  All  71  4 weeks | Tofacitinib 2% 2 twice/day, 2% twice/day, vehicle twice/2 twice/day | 1/efficacy  Target Plaque Severity Score (TPSS) Change in target lesion area | Pfizer  11  2  EEUU |
| Tofacitinib  Topical  NCT00678561 | N=81  2a  10-2008  07-2009 | 1 | 18 years and older  All  81  4 weeks | Tofacitinib 2%,0,2%,0,02% QD, 2%,0,2%,0,02% BID  Placebo | 1/efficacy  Target plaque severity score (TPSS) | Pfizer  19  2  EEUU |
| Tofacitinib  Topical  NCT01831466 | N=476  2b  05-2013  09-2014 | 1 | 18 years and older  All  476  12 weeks | Tofacitinib 1% QD, 2% QD, 1% BID, 2%BID  /Placebo | 2/efficacy  PGA 0-1 | Pfizer  54  4  EEUU |
| Ruxolitinib  Topical  NCT00820950 | N=29  **2**  05-2007  05-2009 | 1 | 18-75 years old  All  29  8weeks | Ruxolitinib 0,5%, 1%, 1,5% cream QD or BID, Placebo, calcipotriene, betamethasone dipropionate | 1/efficacy/  Change in target lesion scores.  Change in target lesión area | Incyte Coorporation  6  1  EEUU |
| Ruxolitinib  Topical  NCT00617994 | N=25  2  08-2007  02-2009 | 0 | 18-65 years old  All  25  28 days | Ruxolitinib 1,5% cream BID, | 1/safety/  Adverse experiences, measuring VS and ECGs, clinical laboratory blood and urine samples | Incyte Coorporation  4  1  EEUU |
| Ruxolitinib  Topical  NCT00778700 | N=199  2  08-2008  04-2009 | 0 | 18-75 years old  All  199  84 days | Ruxolitinib  0.5%,1%,1.5% cream. Placebo | 1/efficacy  Absolute change from Baseline to Day 84 in total lesion score for all treatable psoriatic lesions. | Incyte Coorporation  28  1  EEUU |
| Baricitinib  Systemic  NCT01490632 | N=271  2b  12-2011  08-2014 | 2 | 18 years and older  All  271  12-52 weeks | Baricitinib 2mg, 4 mg, 8 mg, 10 mg daily, placebo | 1/efficacy/PASI75 | Eli Lilly and Company/Incyte Coorporation  35  3  EEUU |
| Peficitinib  Systemic  NCT01096862 | N=124  2a  03-2010  07-2011 | 2 | 18 years and older  All  124  6 weeks | Peficitinib  Low, lowest, medium, high, highest dose  Placebo | 2/efficacy-safety/PASI 75 | Astellas  11  1  EEUU |
| Solcitinib  Systemic  NCT01782664 | N=68  2  03-2013  03-2014 | 1 | 18-75 years old  All  68  12 weeks | Solcitinib 100mg, 200mg, 400mg  Placebo | 2/efficacy/PASI 75 | GlaxoSmithKline  15  2  Germany |
| Abrocitinib  Systemic  NCT02201524 | N=59  2  11-2014  09-2015 | 1 | 18-65 years old  All  59  4 weeks | PF 04965842 200 mg one day, 200 mg twice day, 400 mg one day, placebo | 1/efficacy/PASI | Pfizer  45  2  EEUU |
| Itacitinib  Systemic  NCT01634087 | N=50  2  06-2012  02-2013 | 1 | 18-75 years old  All  50  28 days | Itacitinib 100mg QD/BID, 200mg QD/BID, 600mg QD/BID, placebo | 2/safety-efficacy/ Safety and tolerability measured by changes in frequency and severity of adverse events, ECGs, physical examination, vital signs, and clinical laboratory | Incyte coorporation  9  2  EEUU |
| Deucravacitinib  Systemic  NCT02931838 | N=268  2  11-2016  11-2017 | 1 | 18-70 years old  All  12 weeks | Deucravacitinib (5 doses)  placebo | 1 efficacy PASI75/ 1 safety | Bristol-Myers Squibb  76  8 |
| Deucravacitinib  Systemic  NCT03924427 | N=80  3  04-2019  11-2020 | 0 | >20 years old  All  16 weeks | Deucravacitinib | 2/efficacy PGA 0-1/ PASI 75 | Bristol-Myers Squibb  27  1 |
| Deucravacitinib  Systemic  NCT04772079 | N=84  3  03-2021  04-2024 | 0 | 12-18 years old  All  2-16 weeks | Deucravacitinib (2 doses),placebo | 5/efficacy PGA/ PASI/ pharmacokinetics data | Bristol-Myers Squibb  16  8 |
| Deucravacitinib  Systemic  NCT04036435 | N=1680  3  01-2020  01-2024 | 0 | All  All  96 weeks | Deucravacitinib | 1/ safety/ Incidence of Adverse Events (AE) and Serious Adverse Events (SAE) | Bristol-Myers Squibb  314  20 |
| Deucravacitinib  Systemic  NCT03624127 | N=666  3  01-2020  01-2024 | 0 | All  All  96 weeks | Deucravacitinib  Placebo  Apremilast | 2 /efficacy/  PGA/PASI | Bristol-Myers Squibb  165  11 |
| Deucravacitinib  Systemic  NCT04167462 | N=180  3  11-2019  01-2022 | 0 | >18 years old  All  16 weeks | Deucravacitinib  Placebo  a | 2 /efficacy/  PGA 0-1/PASI 75 | Bristol-Myers Squibb  36  3 |
| Deucravacitinib  Systemic  NCT03611751 | N=1000  3  7-2018  12-2020 | 0 | >18 years old  All  16 weeks | Deucravacitinib  Placebo  Apremilast | 2 /efficacy/  PGA 0-1/PASI 75 | Bristol-Myers Squibb  205  15 |
| Deucravacitinib  Systemic  NCT02534636 | N=140  1  10-2015  11-2016 | 0 | 18 -70 years old  All  12 weeks | Deucravacitinib  Placebo  Famotidine  Intherpheron 2 alpha recombinant | 4/ safety/ Adverse Event (AE), Serious adverse event (SAE) | Bristol-Myers Squibb  1  1 |
| Brepocitinib  Systemic  NCT02969018 | N=212  2  12-2016  03-2018 | 1 | 18-75 years old  All  12 weeks | Brepocitinib 30 mg BID, 30 mg QD, 60 mg QD, 60 mg BID;  Placebo | 1/efficacy /PASI 75 | Pfizer  3  44 |
| Brepocitinib  Topical  NCT03850483 | N=240  2  04-2019  04-2021 | 0 | 18-75 years old  All  12 weeks | Brepocitinib, 0,1% 0,3%, 1%;3%;  placebo | 1/efficacy/PASI 75 | Pfizer  79  10 |
| Brepocitinib  Systemic  NCT02310750 | N=96  1  11-2014  02-2016 | 1 | 18-65 years old  All  1-4 weeks | Brepocitinib  placebo | 22/ pharmacokinetic-safety/ Change From Baseline in Blood Pressure at Day 1 | Pfizer  1  1 |

**Table S2. Tofacitinib systemic treatment efficay and safety.**

*24 weeks data, BID; oral twice daily; PASI 75: 75% reduction psoriasis area severity index; PGA 01:Physician Global Assessment 01; AE: adverse events; ** Participants received Tofacitinib 10 milligram (mg) tablets orally twice daily from Day 1 until any safety finding requiring study discontinuation (up to a maximum of 67 months); *** 52 weeks; **** Table S2; ***** Indicates events were collected by non-systematic assessment. AE: adverse events

| **Clinical Trial**  **Number/ Study reference** | **Tofacitinib**  **5mg BID**  PASI 75/  PGA01/  12-16 weeks | **Tofacitinib**  **10mg BID**  PASI 75/  PGA01/  12-16  weeks | **Placebo**  PASI75/  PGA01  12-16/  weeks | **Etanercept**  PASI75/  PGA01/  12-16  weeks | **Tofacitinib**  **5mg BID**  AE (12-16) weeks:  Time frame  Total/  Serious/  Most frequent AA | **Tofacitinib**  **10mg BID** AE (12-16 weeks):  Time frame  Total  Serious  Most frequent | **Placebo**  12-16  weeks  AE:  Time  frame  Total  Serious Most frequent | **Etanercept**  12-16  weeks  AE:  Time  Frame/  Total  Serious /Most frequent |
| --- | --- | --- | --- | --- | --- | --- | --- | --- |
| NCT00678210 | 40, 82%  NA  N=49 | NA  NA  NA | 2%  NA  N=50 | NA  NA  NA | Not specified  28  1  N=48  Upper respiratory  tract  infection  ^*****^ | Not specified  NA  NA  NA  ^*****^ | Not specified  30  0  N=50  Upper  respiratory  tract  infection  ^*****^ | NA NA NA |
| NCT01186744 | 38,07%  37,16%  N=331 | 60,60%  58,81%  N=335 | NA  NA  N=107 | NA NA | Not specified*  101  6  N=218  Nasopha-  Ryngitis  ^*****^ | Not specified*  94  9  N=157  Nasopha-  Ryngitis  ^*****^ | Not specified  NA  NA  NA  ^*****^ | NA NA |
| NCT01241591 | 39,51%  47,11%  N=329 | 63,64%  68,18%  N=330 | 5,61%  14,95%  N=107 | 58,81%  66,27%  n=335 | Not specified  103  7  Nasopha-  Ryngitis  ^*****^ | Not specified  121  5  Nasopha-  Ryngitis  ^*****^ | Not specified  34  2  Nasopha-  Ryngitis  ^*****^ | Not specified  109  7  Nasopha-  Ryngitis  ^*****^ |
| NCT01276639 | 39,94%  41,87%  N=363 | 59,17%  59,17%  N=360 | 6,21%  9,04%  N=177 | NA  NA | Not specified^***^  39  189  24  Nasopha-  Ryngitis  ^*****^ | Not specified^***^  9  222  19  Nasopha-  Ryngitis  ^*****^ | Not specified^***^  34  2  Nasopha-  Ryngitis  ^*****^ | NA NA NA |
| NCT01309737 | 46,01%  46,10%  N=376 | 59,63%  59,90%  N=374 | 11,40%  10,88%  N=193 | NA  NA  NA | Not specified^***^  207  18  N=382  Nasopha-  Ryngitis  ^*****^ | Not specified^***^  213  19  N=381  Nasopha-  Ryngitis  ^*****^ | Not specified^***^  113  12  N=193  Nasopha-  Ryngitis  ^*****^ | NA  NA  NA |
| NCT01710046 | NA  NA  NA | 62,5%  50%  N=8 | 33,3%  33,3%  N=3 | NA  NA  NA | NA  NA  NA | 16 weeks  12  2  0  Nasopha-  Ryngitis  ^*****^ | 16 weeks  1  1  Gravitational oedema  ^*****^ | NA  NA  NA |
| NCT01519089 | 62,80%  67,4%  N=43 | 72,7%  68,2%  N=44 | NA  NA  NA | NA  NA  NA | Not specified^***^  27  0  Nasopha-  Ryngitis  ^*****^ | Not specified^***^  28  1  Nasopha-  Ryngitis  ^*****^ | NA  NA  NA  ^*****^ | NA  NA |
| NCT01163253 | NA  NA  NA | 54,79%  58,45%  N=2200 | NA  NA  NA | NA  NA  NA | NA  NA  NA | Not specified (max 67 months)  1415  304  Nasopha-  Ryngitis  ^*****^ | NA  NA  NA | NA  NA  NA |
| Serum Human Beta-Defensin-2  [21^]****^ | 80%  NA  N=5 | 100%  NA  N=7 | 0%  NA  N=6 | NA  NA  NA | 2  0  Nasopha-  Ryngitis | 2  0  Nasopha-  Ryngitis | 2  0  Hyperlipidaemia | NA  NA  NA |
| NCT01815424 | 54,6%  52,3%  N=88 | 81,10%  75,60%  N=90 | 12,5%  19,30%  N=88 | NA  NA  NA | 52 weeks  60  4  Upper respiratoy tract infecction ^*****^ | 52 weeks  59  2  Upper  respiratory tract  infecction^*****^ | 52 weeks  23  0  Hyperlipi  daemia^*****^ | NA  NA  NA |
| NCT01736696 | NA  NA  NA | NA  NA  NA | NA  NA  NA | NA  NA  NA | Not specified 14 days  0  0  NA  ^*****^ | Not specified 14 days  1  1  Headache  ^*****^ | Not specified 14 days  2  0  Headache  ^*****^ | NA  NA |

**Table S3. Summary of efficacy and safety of the use of the different anti-JAK drugs in skin psoriasis.**

Abbreviations: PASI 75:75% reduction Psoriasis Area Severity Index, PGA01: Physician global assessment 01,PGAs change* NDA: No data available. QD: once daily, BID: twice daily

| **Clinical trial** | **Phase** | **N** | **Drugs/dose** | **Primary**  **enpoint** | **PASI 75** | **PGA01** | **AE** |
| --- | --- | --- | --- | --- | --- | --- | --- |
| NCT00678210 | 2 | 49 | Tofacitinib 5 mg BID | 12 weeks | 40,82% | NDA | Upper respiratoy tract infection |
| NCT01186744 | 3 | 331 | Tofacitinib  5 mg BID | 16 weeks | 38,07% | 37,16% | Nasopha-ryngitis |
|  |  | 335 | Tofacitinib  10 mg BID | 16 weeks | 60,60% | 58,81% | Nasopha-ryngitis |
| NCT01241591 | 3 | 329 | Tofacitinib  5mg BID | 12 weeks | 39,51% | 47,11% | Nasopha-ryngitis |
|  |  | 330 | Tofacitinib 10 mg BID | 12  weeks | 63,64% | 68,18% | Nasopha-ryngitis |
| NCT01276639 | 3 | 363 | Tofacitinib  5mg BID | 16  weeks | 39,94% | 41,87% | Nasopha-ryngitis |
|  |  | 360 | Tofacitinib 10 mg BID | 16  weeks | 59,17% | 59,17% | Nasopha-ryngitis |
| NCT01309737 | 3 | 376 | Tofacitinib  5mg BID | 16 weeks | 46,01% | 46,10% | Nasopha-ryngitis |
|  |  | 374 | Tofacitinib  5mg BID | 16  weeks | 59,63% | 59,90% | Nasopha-ryngitis |
| NCT01710046 | 2a | 8 | Tofacitinib  10 mg BID | 12  weeks | 62,5% | 50% | Nasopha-ryngitis |
| NCT01519089 | 3 | 43 | Tofacitinib  5mg BID | 16 weeks | 62,80% | 67,4% | Nasopha-ryngitis |
|  |  | 44 | Tofacitinib  10 mg BID | 16 weeks | 72,7% | 68,2% | Nasopha-ryngitis |
| NCT01163253 | 3 | 2220 | Tofacitinib  10 mg BID | 12 weeks | 54,79% | 58,45% | Nasopha-ryngitis |
| Serum Human BetaDefensin2 | [21^]****^ | 5 | Tofacitinib  5mg BID | 16  weeks | 80% | NDA | Nasopha-ryngitis |
|  |  | 7 | Tofacitinib  10 mg BID | 16  weeks | 100% | NDA | Nasopha-ryngitis |
| NCT01815424 | 3 | 88 | Tofacitinib  5mg BID | 16 weeks | 54,6% | 52,3% | Upper respiratoy tract infection |
|  |  | 90 | Tofacitinib  10 mg BID | 16  weeks | 81,10% | 75,6% | Upper respiratoy tract infection |
| NCT01736696 | 1 |  | Tofacitinib  5mg BID | 14 days | NDA | NDA | NA |
|  |  |  | Tofacitinib  10 mg BID | 14 days | NDA | NDA | Headache |
| NCT01096862 | 2a | 19 | Peficitinib  10 mg BID | 6 weeks | 31,6% | NDA | Nasal congestion |
|  |  | 21 | Peficitinib  25 mg BID | 6 weeks | 14,3% | NDA | Dry mouth |
|  |  | 19 | Peficitinib  60 mg BID | 6 weeks | 57,9% | NDA | Nasopha-ryngitis |
|  |  | 17 | Peficitinib  100mgBID | 6 weeks | 64,7% | NDA | Nasopha-ryngitis |
|  |  | 19 | Peficitinib  50 mg QD | 6  weeks | 15,8% | NDA | Headache |
| NCT01490632 | 2b | 28 | Baricitinib  2 mg QD | 12  weeks | 28,6% | NDA | Fatigue |
|  |  | 63 | Baricitinib  4 mg QD | 12  weeks | 28,6% | NDA | Urinary tract infection |
|  |  | 56 | Baricitinib  8 mg QD | 12  weeks | 42,9% | NDA | Nasopha-ryngitis |
|  |  | 61 | Baricitinib  10 mg QD | 12  weeks | 54,1% | NDA | Nasopha-ryngitis |
| NCT01782664 | 2b | 15 | Solcitinib  100 mg | 12  weeks | 13% | NDA | Headache |
|  |  | 16 | Solcitinib  200 mg | 12  weeks | 25% | NDA | Nasopha-ryngitis |
|  |  | 14 | Solcitinib  400 mg | 12  weeks | 57% | NDA | Nasopha-ryngitis |
| NCT01634087 | 2 | 9 | Itacitinib  100mgQD | 28  days | NDA | -22,3%  * | Nasopha-ryngitis |
|  |  | 9 | Itacitinib  200mgQD | 28  days | NDA | -29,4%  * | Nasopha-ryngitis |
|  |  | 9 | Itacitinib  200mgBID | 28  days | NDA | -35,2%  * | Nasopha-ryngitis |
|  |  | 11 | Itacitinib  600mgBID | 28  days | NDA | -42,4%  * | Nasopha-ryngitis |
| NCT02931838 | 2 | 44 | Deucravacitinib  6 mg BID | 12  weeks | 75% | 75% | Blood creatine phosphokinase increased |
|  |  | 44 | Deucravacitinib  6 mg QD | 12  weeks | 66,7% | 64,4% | ryngitis |
|  |  | 45 | Deucravacitinib  3 mg BID | 12  weeks | 68,9% | 75,6% | Nasopha-ryngitis |
|  |  | 45 | Deucravacitinib  3 mg QOD | 12  weeks | 38,6% | 38,6% | Nasopha-ryngitis |
|  |  | 44 | Deucravacitinib  3 mg QD | 12  weeks | 9,1% | 20,5% | Headache  Nausea |
| NCT03924427 | 3 | NDA | Deucravacitinib | 16 weeks | NDA | NDA | NDA |
| NCT04772079 | 3 | NDA | Deucravacitinib | 2  weeks | NDA | NDA | NDA |
| NCT04036435 | 3 | NDA | Deucravacitinib | 244  weeks | NDA | NDA | NDA |
| NCT03624127 | 3 | NDA | Deucravacitinib | 16  weeks | NDA | NDA | NDA |
| NCT04167462 | 3 | NDA | Deucravacitinib | 16  weeks | NDA | NDA | NDA |
| NCT03611751 | 3 | NDA | Deucravacitinib | 16  weeks | NDA | NDA | NDA |
| NCT02534636 | 1 | NDA | Deucravacitinib | 12  weeks | NDA | NDA | NDA |
| NCT02310750 | 1 | 14 | Brepocitinib  30 mg QD | 4  weeks | NDA | NDA | Blood creatinine increased |
|  |  | 9 | Brepocitinib  100mg QD | 4  weeks | NDA | NDA | Blood creatinine increased |
| NCT02969018 | 2 | 25 | Brepocitinib  30 mg QD | 12  weeks | 86% | NDA | nasopharyngitis |
|  |  | 19 | Brepocitinib  30mgQD* 4 weeks followed 100 mg once weekely | 12  weeks | 36,7% | NDA | nasopharyngitis |
| NCT03850483 | 2 |  | Brepocitinib | 12 weeks | NDA | NDA | NDA |
