# Supplementary material for: A Scoping Review on Use of Drugs Targeting the JAK/STAT Pathway in Psoriasis
Source: Front Med (Lausanne). 2022 Feb 25;9:754116. doi: 10.3389/fmed.2022.754116 (PMC8914468; doi:10.3389/fmed.2022.754116)
Supplement: Supplementary file 2 [file Data_Sheet_2.docx]

SUPPLEMENTARY MATERIAL

**Literature Search**

We performed a three-step literature search approach to identify and locate all relevant studies on the use of drugs targeting the JAK pathway in the treatment of dermatological diseases. Later, we selected articles related to the use of these drugs in patients with psoriasis. The first step, performed on October 4^th^ 2018, entailed an initial limited search of the MEDLINE and EMBASE databases using the following [search query: ('janus kinase inhibitor'/exp OR 'janus kinase inhibitor') AND ('skin disease' OR 'psoriasis' OR 'vitiligo' OR 'alopecia' OR 'dermatitis' OR 'atopic dermatitis'), and the subsequent analysis of the text contained in the titles, abstracts and index terms which described the articles. The second step consisted of a new search of the same databases on October 29, 2018 but using a more complex strategy derived from the keywords we previously extracted (Table S1). Results were ordered by disease, based on title and abstract review. Subsequently, sources related to psoriasis were selected. Lastly, we searched the reference list of all selected reports and articles for additional relevant studies. Authors of primary studies or reviews were contacted for further information when necessary. This process was performed independently by two researchers (AM and FG-G) and further updated to include studies published up to April 2019. Finally, a study search was conducted on ClinicalTrials.gov with previously anti-jak drugs used in plaque psoriasis in March 2021.

**Selection of información sources**

Where possible data were extracted fron de ClinicalTrials.gov. When they were not available the data published in scientific journals and conference abstracts were used in this order,

SUPPLEMENTARY TABLES

**Table S1.** Mapping studies

**Table S2.** Tofacitinib systemic treatment efficacy and safety.

**Table S3.** Summary of efficacy and safety of the use of the different anti-JAK drugs in skin psoriasis.

**Table S4.** Search strategies in literature databases.

**Table S5.** List of included studies.

**Table S6.** List of excluded studies with reasons for exclusion.

**Table S7.** Metadata article-references

**Table S8.** Serious AE systemic Tofacitinib

**Table S9.** Efficacy/safety topical Tofacitinib oinment

**Table S10.** Efficacy/safety topical ruxolitinib ointment

**Table S11.** Efficacy/safety peficitinib treatment

**Table S12.** Baricitinib efficacy/safety

**Table S13.** Serious baricitinib AE

**Table S14.** Solcitonib efficacy/safety

**Table S15.** Solcitinib serious AE

**Table S16.** Itacitinib efficacy/safety

**Table S17.** Itacitinib serious AE

**Table S18.** Deucravacitanib**/**BMS-986165 efficacy/safety

**Table S19.** Abrocitinib PF-04965842 efficacy/safety

**Table S20.** PF-06700841 efficacy/safety

Note.

In tables methodology adverse events assessment are defined: :

“Systematic Assessment”: Any method of routinely determining whether or not certain adverse events have occurred, for example through a standard questionnaire, regular investigator assessment, regular laboratory testing, or other method.

“Non-Systematic Assessment”: Any non-systematic method for determining whether or not adverse events have occurred, such as self-reporting by participants or occasional assessment / testing.

**Table S4. Search strategies in Web of Science database.**

| **Search** | **Results** | **Strategy** |
| --- | --- | --- |
| # 5 | **1.217** | #4 AND #1  *Bases de datos= WOS Período de tiempo=Todos los años*  *Idioma de búsqueda=Auto* |
| # 4 | **988.291** | #3 OR #2  *Bases de datos= WOS Período de tiempo=Todos los años*  *Idioma de búsqueda=Auto* |
| # 3 | **664.079** | **TEMA:** (skin disease) *OR* **TEMA:** (rosaceae) *OR* **TEMA:** (scleroderma) *OR* **TEMA:** (cinca syndrome) *OR* **TEMA:** (hyperhidrosis) *OR* **TEMA:**(erythropoietic protoporphyria) *OR* **TEMA:** (anca associated vasculitis) *OR* **TEMA:** (seborrheic dermatitis) *OR* **TEMA:** (herpes) *OR* **TEMA:**(sjoegren syndrome)  *Bases de datos= WOS Período de tiempo=Todos los años*  *Idioma de búsqueda=Auto* |
| # 2 | **432.287** | **TEMA:** (psoriasis) *OR* **TEMA:** (atopic dermatitis) *OR* **TEMA:** (alopecia) *OR* **TEMA:** (contact dermatitis) *OR* **TEMA:** (vitiligo) *OR* **TEMA:**(graft versus host reaction) *OR* **TEMA:** (lichen planus) *OR* **TEMA:** (pyoderma gangrenosum) *OR* **TEMA:** (pruritus) *OR* **TEMA:** (eosinofilic annulare erythema) *OR* **TEMA:** (male type alopecia) *OR* **TEMA:** (proteasome associated autoinflammatory syndrome) *OR* **TEMA:** (sting associated vasculopathy with onset in infancy) *OR* **TEMA:** (chronic atypical neutrophilic dermatosis with lipodystrophy and elevated temperature syndrome) *OR* **TEMA:** (hand dermatitis) *OR* **TEMA:** (discoid lupus erythematosus) *OR* **TEMA:** (mucocutaneous candidiasis) *OR* **TEMA:** (urticaria) *OR* **TEMA:** (suppurative hidradenitis) *OR* **TEMA:** (melanoma) *OR* **TEMA:** (non melanoma skin cancer) *OR* **TEMA:** (acne) *OR* **TEMA:** (lichen sclerosus) *OR* **TEMA:** (pityriasis rubra pilaris) *OR* **TEMA:** (pemphigus)  *Bases de datos= WOS Período de tiempo=Todos los años*  *Idioma de búsqueda=Auto* |
| # 1 | **10.669** | **TEMA:** (tofacitinib) *OR* **TEMA:** (baricitinib) *OR* **TEMA:** (ruxolitinib) *OR* **TEMA:** (oclacitinib) *OR* **TEMA:** (upadacitinib) *OR* **TEMA:**(delgocitinib) *OR* **TEMA:** (itacitinib) *OR* **TEMA:** (momelotinib) *OR* **TEMA:** (peficitinib) *OR* **TEMA:** (decernotinib) *OR* **TEMA:** (fedratinib) *OR***TEMA:** (pacritinib) *OR* **TEMA:** (filgotinib) *OR* **TEMA:** (gandotinib) *OR* **TEMA:** (solcitinib) *OR* **TEMA:** (lestaurtinib) *OR* **TEMA:** (janus kinase inhibitor)  *Bases de datos= WOS Período de tiempo=Todos los años*  *Idioma de búsqueda=Auto* |

**Database**: SCOPUS

| ((TITLE-ABS-KEY (tofacitinib) OR TITLE-ABS-KEY (baricitinib) OR TITLE-ABS-KEY (ruxolitinib) OR TITLE-ABS-KEY(oclacitinib) OR TITLE-ABS-KEY(upadacitinib) OR TITLE-ABS-KEY(delgocitinib) OR TITLE-ABS-KEY(itacitinib) OR TITLE-ABS-KEY(momelotinib) OR TITLE-ABS-KEY(peficitinib) OR TITLE-ABS-KEY(decernotinib) OR TITLE-ABS-KEY(fedratinib) OR TITLE-ABS-KEY(pacritinib) OR TITLE-ABS-KEY(filgotinib) OR TITLE-ABS-KEY(gandotinib) OR TITLE-ABS-KEY(solcitinib) OR TITLE-ABS-KEY(lestaurtinib) OR TITLE-ABS-KEY(janus kinasa inhibitor))) AND ( ((TITLE-ABS-KEY(psoriasis) OR TITLE-ABS-KEY(atopic dermatitis) OR TITLE-ABS-KEY(alopecia) OR TITLE-ABS-KEY(contact dermatitis) OR TITLE-ABS-KEY(vitiligo) OR TITLE-ABS-KEY(graft versus host reaction) OR TITLE-ABS-KEY(lichen planus) OR TITLE-ABS-KEY(pyoderma gangrenosum) OR TITLE-ABS-KEY(pruritus) OR TITLE-ABS-KEY(eosinofilic annulare erythema) OR TITLE-ABS-KEY(male type alopecia) OR TITLE-ABS-KEY(proteasome associated autoinflammatory syndrome) OR TITLE-ABS-KEY(sting associated vasculopathy with onset in infancy) OR TITLE-ABS-KEY(chronic atypical neutrophilic dermatosis with lipodystrophy and elevated temperature syndrome) OR TITLE-ABS-KEY(hand dermatitis) OR TITLE-ABS-KEY(discoid lupus erythematous) OR TITLE-ABS-KEY(mucocutaneous candidiasis) OR TITLE-ABS-KEY(suppurative hidradenitis) OR TITLE-ABS-KEY(melanoma) OR TITLE-ABS-KEY(non melanoma skin cancer) OR TITLE-ABS-KEY(acne) OR TITLE-ABS-KEY(lichen sclerosus) OR TITLE-ABS-KEY(pityriasis rubra pilaris) OR TITLE-ABS-KEY(pemphigus) OR TITLE-ABS-KEY(skin disease) OR TITLE-ABS-KEY(rosaceae) OR TITLE-ABS-KEY(scleroderma) OR TITLE-ABS-KEY(cinca syndrome) OR TITLE-ABS-KEY(hyperhidrosis) OR TITLE-ABS-KEY(erythropoietic protoporphyria) OR TITLE-ABS-KEY(anca associated vasculitis))) or ((TITLE-ABS-KEY(seborrheic dermatitis) OR TITLE-ABS-KEY(herpes simplex)OR TITLE-ABS-KEY(sjogren syndrome)))) |
| --- |

**Database**: MEDLINE

| ('psoriasis'/exp OR psoriasis OR 'atopic dermatitis' OR 'alopecia' OR 'contact dermatitis' OR 'vitiligo' OR 'graft versus host reaction' OR 'lichen planus' OR 'pyoderma gangrenosum' OR 'pruritus' OR (eosinofilic AND annulare AND erythema) OR 'male type alopecia' OR 'proteasome associated autoinflammatory syndrome' OR 'sting associated vasculopathy with onset in infancy' OR 'chronic atypical neutrophilic dermatosis with lipodystrophy and elevated temperature syndrome' OR 'hand dermatitis' OR 'discoid lupus erythematosus' OR 'mucocutaneous candidiasis' OR (urticaria AND chronic) OR 'suppurative hidradenitis' OR 'melanoma' OR 'non melanoma skin cancer' OR 'acne' OR 'lichen sclerosus et atrophicus' OR 'pityriasis rubra pilaris' OR 'pemphigus' OR 'skin disease' OR 'rosaceae' OR 'scleroderma' OR 'cinca syndrome' OR 'hyperhidrosis' OR 'erythropoietic protoporphyria' OR 'anca associated vasculitis' OR 'seborrheic dermatitis' OR 'herpes simplex' OR 'sjoegren syndrome') AND ('tofacitinib' OR 'baricitinib' OR 'ruxolitinib' OR 'oclacitinib' OR 'upadacitinib' OR 'delgocitinib' OR 'itacitinib' OR 'momelotinib' OR peficitinib OR 'decernotinib' OR 'fedratinib' OR 'pacritinib' OR 'filgotinib' OR 'gandotinib' OR 'solcitinib' OR 'lestaurtinib' OR 'janus kinase inhibitor') AND [4-10-2018]/sd NOT [26-4-2019]/sd |
| --- |

**ClinicalTrials.gov**

| 1.-Tofacitinib and psoriasis/2.-Ruxolitinib and psoriasis/3.-peficitinib and psoriasis/4.-ASP015k and psoriasis/5.-baricitinib and psoriasis/6.-solcitinib and psoriasis/7.-itacitanib and psoriasis/8.-deucravacitanib and psoriasis/9.-BMS986165 and psoriasis/10.-Abrocitinib and psoriasis/11.-PF-04965842 and psoriasis/12.-brepocitinib and psoriasis/13.-PF-06700841 and psoriasis. March 2021 |
| --- |

**Table S5. List of included studies.**

| **ID** | **Article reference** |
| --- | --- |
| 1 | Valenzuela F., Korman N.J., Bissonnette R., Bakos N., Tsai T.-F., Harper M.K., Ports W.C., Tan H., Tallman A., Valdez H., Gardner A.C.Tofacitinib in patients with moderate-to-severe chronic plaque psoriasis: long-term safety and efficacy in an open-label extension study. British Journal of Dermatology (2018) 179:4 (853-862). Date of Publication: 1 Oct 2018 |
| 2 | Fitz L., Zhang W., Soderstrom C., Fraser S., Lee J., Quazi A., Wolk R., Mebus C.A., Valdez H., Berstein G.Association between serum interleukin-17A and clinical response to tofacitinib and etanercept in moderate to severe psoriasis. Clinical and Experimental Dermatology (2018) 43:7 (790-797). Date of Publication: 1 Oct 2018 |
| 3 | Ma G., Xie R., Strober B., Langley R., Ito K., Krishnaswami S., Wolk R., Valdez H., Rottinghaus S., Tallman A., Gupta P.Pharmacokinetic Characteristics of tofacitinib in Adult Patients With Moderate to Severe Chronic Plaque Psoriasis. Clinical Pharmacology in Drug Development (2018) 7:6 (587-596). Date of Publication: 1 Aug 2018 |
| 4 | Schmieder G.J., Draelos Z.D., Pariser D.M., Banfield C., Cox L., Hodge M., Kieras E., Parsons-Rich D., Menon S., Salganik M., Page K., Peeva E.Efficacy and safety of the Janus kinase 1 inhibitor PF-04965842 in patients with moderate-to-severe psoriasis: phase II, randomized, double-blind, placebo-controlled study. British Journal of Dermatology (2018) 179:1 (54-62). Date of Publication: 1 Jul 2018 |
| 5 | Winthrop K.L., Korman N., Abramovits W., Rottinghaus S.T., Tan H., Gardner A., Mukwaya G., Kaur M., Valdez H. T-cell–mediated immune response to pneumococcal conjugate vaccine (PCV-13) and tetanus toxoid vaccine in patients with moderate-to-severe psoriasis during tofacitinib treatment. Journal of the American Academy of Dermatology (2018) 78:6 (1149-1155.e1). Date of Publication: 1 Jun 2018 |
| 6 | Soriano E.R., Madariaga H., Castañeda O., Citera G., Schneeberger E.E., Cardiel M.H., Hendrikx T., Graham D., Shi H., Ponce De Leon D. Liver enzyme abnormalities after tofacitinib treatment in patients with hepatic steatosis from the rheumatoid arthritis, psoriatic arthritis and psoriasis clinical programmes Annals of the Rheumatic Diseases (2018) 77 Supplement 2 (593-594). Date of Publication: 1 Jun 2018 |
| 7 | Mease P.J., Kremer J., Cohen S., Curtis J.R., Charles-Schoeman C., Loftus E.V., Greenberg J., Palmetto N., Kanik K.S., Graham D., Wang C., Biswas P., Chan G., DeMasi R., Valdez H., Hendrikx T., Jones T.V. Incidence of thromboembolic events in the tofacitinib rheumatoid arthritis, psoriasis, psoriatic arthritis and ulcerative colitis development programmes. Annals of the Rheumatic Diseases (2018) 77 Supplement 2 (983). Date of Publication: 1 Jun 2018 |
| 8 | Kuo C.-M., Tung T.-H., Wang S.-H., Chi C.-C. Efficacy and safety of tofacitinib for moderate-to-severe plaque psoriasis: a systematic review and meta-analysis of randomized controlled trials. Journal of the European Academy of Dermatology and Venereology (2018) 32:3 (355-362). Date of Publication: 1 Mar 2018 |
| 9 | Dasic G., Jones T., Frajzyngier V., Rojo R., Madsen A., Valdez H. Safety signal detection and evaluation in clinical development programs: A case study of tofacitinib. Pharmacology Research and Perspectives (2018) 6:1 Article Number: e00371. Date of Publication: 1 Feb 2018 |
| 10 | Kim J., Tomalin L., Lee J., Fitz L.J., Berstein G., Correa-da Rosa J., Garcet S., Lowes M.A., Valdez H., Wolk R., Suarez-Farinas M., Krueger J.G. Reduction of Inflammatory and Cardiovascular Proteins in the Blood of Patients with Psoriasis: Differential Responses between tofacitinib and Etanercept after 4 Weeks of Treatment. Journal of Investigative Dermatology (2018) 138:2 (273-281). Date of Publication: 1 Feb 2018 |
| 11 | Ständer S., Luger T.A., Cappelleri J.C., Bushmakin A.G., Mamolo C., Zielinski M.A., Tallman A.M., Yosipovitch G. Efficacy of Systemic Treatments of Psoriasis on Pruritus: A Systemic Literature Review and Meta-Analysis. Journal of Investigative Dermatology (2018) 138:1 (38-45). Date of Publication: 1 Jan 2018 |
| 12 | Ständer S., Luger T.A., Cappelleri J.C., Bushmakin A.G., Mamolo C., Zielinski M.A., Tallman A.M., Yosipovitch G. Validation of the itch severity item as a measurement tool for pruritus in patients with psoriasis: Results from a phase 3 tofacitinib program. Acta Dermato-Venereologica (2018) 98:3 (340-345). Date of Publication: 2018 |
| 13 | Strober B.E., Gottlieb A.B., van de Kerkhof P.C.M., Puig L., Bachelez H., Chouela E., Imafuku S., Thaçi D., Tan H., Valdez H., Gupta P., Kaur M., Frajzyngier V., Wolk R. Benefit–risk profile of tofacitinib in patients with moderate-to-severe chronic plaque psoriasis: pooled analysis across six clinical trials. British Journal of Dermatology (2018). Date of Publication: 2018 |
| 14 | Huang F., Luo Z.-C. Adverse drug events associated with 5mg versus 10mg tofacitinib (Janus kinase inhibitor) twice daily for the treatment of autoimmune diseases: A systematic review and meta-analysis of randomized controlled trials. Clinical Rheumatology (2018). Date of Publication: 2018 |
| 15 | Sbidian E., Chaimani A., Garcia-Doval I., Do G., Hua C., Mazaud C., Droitcourt C., Hughes C., Ingram J.R., Naldi L., Chosidow O., Le Cleach L. Systemic pharmacological treatments for chronic plaque psoriasis: A network meta-analysis. Cochrane Database of Systematic Reviews (2017) 2017:12 Article Number: CD011535. Date of Publication: 22 Dec 2017 |
| 16 | Checchio T., Ahadieh S., Gupta P., Mandema J., Puig L., Wolk R., Valdez H., Tan H., Krishnaswami S., Tallman A., Kaur M., Ito K. Quantitative Evaluations of Time-Course and Treatment Effects of Systemic Agents for Psoriasis: A Model-Based Meta-Analysis. Clinical Pharmacology and Therapeutics (2017) 102:6 (1006-1016). Date of Publication: 1 Dec 2017 |
| 17 | Abe M., Nishigori C., Torii H., Ihn H., Ito K., Nagaoka M., Isogawa N., Kawaguchi I., Tomochika Y., Kobayashi M., Tallman A.M., Papp K.A. Tofacitinib for the treatment of moderate to severe chronic plaque psoriasis in Japanese patients: Subgroup analyses from a randomized, placebo-controlled phase 3 trial. Journal of Dermatology (2017) 44:11 (1228-1237). Date of Publication: 1 Nov 2017 |
| 18 | Kong B.Y., Immaneni S., Woodruff T.K., Paller A.S., Xu S. The potential impact on future fertility for biologics and emerging therapies for psoriasis and atopic dermatitis. Journal of Investigative Dermatology (2017) 136:10 (B4). Date of Publication: 1 Oct 2017 |
| 19 | Zhang J., Tsai T.-F., Lee M.-G., Zheng M., Wang G., Jin H., Gu J., Li R., Liu Q., Chen J., Tu C., Qi C., Zhu H., Ports W.C., Crook T. The efficacy and safety of Tofacitinib in Asian patients with moderate to severe chronic plaque psoriasis: A Phase 3, randomized, double-blind, placebo-controlled study. Journal of Dermatological Science (2017) 88:1 (36-45). Date of Publication: 1 Oct 2017 |
| 20 | Bachelez H., Griffiths C.E.M., Papp K., Hall S., Merola J.F., Feldman S.R., Khraishi M., Tallman A., Tan H., Hsu M.-A. Effect of tofacitinib on efficacy and patient-reported outcomes in psoriasis patients with baseline psoriatic arthritis: A pooled analysis of 2 phase 3 studies. Arthritis and Rheumatology (2017) 69 Supplement 10. Date of Publication: 1 Oct 2017 |
| 21 | Jin T., Sun Z., Chen X., Wang Y., Li R., Ji S., Zhao Y.Serum Human Beta-Defensin-2 Is a Possible Biomarker for Monitoring Response to JAK Inhibitor in Psoriasis Patients. Dermatology (2017) 233:2-3 (164-169). Date of Publication: 1 Oct 2017 |
| 22 | Wolk R., Armstrong E.J., Hansen P.R., Thiers B., Lan S., Tallman A.M., Kaur M., Tatulych S.Effect of Tofacitinib on lipid levels and lipid-related parameters in patients with moderate to severe psoriasis. Journal of Clinical Lipidology (2017) 11:5 (1243-1256). Date of Publication: 1 Sep 2017 |
| 23 | Brenaut E., Théréné C., Barnetsche T., Misery L.Efficacy of systemic treatments of psoriasis on pruritus: A systemic literature review and meta-analysis. Acta Dermato-Venereologica (2017) 97:8 (1016). Date of Publication: 1 Sep 2017 |
| 24 | Winthrop K.L., Lebwohl M., Cohen A.D., Weinberg J.M., Tyring S.K., Rottinghaus S.T., Gupta P., Ito K., Tan H., Kaur M., Egeberg A., Mallbris L., Valdez H.Herpes zoster in psoriasis patients treated with tofacitinib. Journal of the American Academy of Dermatology (2017) 77:2 (302-309). Date of Publication: 1 Aug 2017 |
| 25 | Merola J.F., Elewski B., Tatulych S., Lan S., Tallman A., Kaur M.Efficacy of tofacitinib for the treatment of nail psoriasis: Two 52-week, randomized, controlled phase 3 studies in patients with moderate-to-severe plaque psoriasis. Journal of the American Academy of Dermatology (2017) 77:1 (79-87.e1). Date of Publication: 1 Jul 2017 |
| 26 | Hutmacher M.M., Papp K., Krishnaswami S., Ito K., Tan H., Wolk R., Valdez H., Mebus C., Rottinghaus S.T., Gupta P.Evaluating Dosage Optimality for tofacitinib, an Oral Janus Kinase Inhibitor, in Plaque Psoriasis, and the Influence of Body Weight. CPT: Pharmacometrics and Systems Pharmacology (2017) 6:5 (322-330). Date of Publication: 1 May 2017 |
| 27 | Mrowietz U., Gerdes S., Gläser R., Schröder O.Successful treatment of refractory alopecia areata universalis and psoriatic arthritis, but not of plaque psoriasis with tofacitinib in a young woman. Acta Dermato-Venereologica (2017) 97:2 (283-284). Date of Publication: 1 Feb 2017 |
| 28 | Griffiths C.E.M., Vender R., Sofen H., Kircik L., Tan H., Rottinghaus S.T., Bachinsky M., Mallbris L., Mamolo C.Effect of tofacitinib withdrawal and re-treatment on patient-reported outcomes: results from a Phase 3 study in patients with moderate to severe chronic plaque psoriasis. Journal of the European Academy of Dermatology and Venereology (2017) 31:2 (323-332). Date of Publication: 1 Feb 2017 |
| 29 | Wang T.-S., Tsai T.-F. Managing Scalp Psoriasis: An Evidence-Based Review. American Journal of Clinical Dermatology (2017) 18:1 (17-43). Date of Publication: 1 Feb 2017 |
| 30 | Tan H., Valdez H., Griffiths C.E.M., Mrowietz U., Tallman A., Wolk R., Gordon K.Early clinical response to tofacitinib treatment as a predictor of subsequent efficacy: Results from two phase 3 studies of patients with moderate-to-severe plaque psoriasis. Journal of Dermatological Treatment (2017) 28:1 (3-7). Date of Publication: 2 Jan 2017 |
| 31 | Feldman S.R., Thaçi D., Gooderham M., Augustin M., de la Cruz C., Mallbris L., Buonanno M., Tatulych S., Kaur M., Lan S., Valdez H., Mamolo C.Tofacitinib improves pruritus and health-related quality of life up to 52 weeks: Results from 2 randomized phase III trials in patients with moderate to severe plaque psoriasis. Journal of the American Academy of Dermatology (2016) 75:6 (1162-1170.e3). Date of Publication: 1 Dec 2016 |
| 32 | Wu J.J., Strober B.E., Hansen P.R., Ahlehoff O., Egeberg A., Qureshi A.A., Robertson D., Valdez H., Tan H., Wolk R.Effects of tofacitinib on cardiovascular risk factors and cardiovascular outcomes based on phase III and long-term extension data in patients with plaque psoriasis. Journal of the American Academy of Dermatology (2016) 75:5 (897-905). Date of Publication: 1 Nov 2016 |
| 33 | Papp K.A., Bissonnette R., Gooderham M., Feldman S.R., Iversen L., Soung J., Draelos Z., Mamolo C., Purohit V., Wang C., Ports W.C.Treatment of plaque psoriasis with an ointment formulation of the Janus kinase inhibitor, tofacitinib: A Phase 2b randomized clinical trial. BMC Dermatology (2016) 16:1 Article Number: 15. Date of Publication: 3 Oct 2016 |
| 34 | Valenzuela F., Paul C., Mallbris L., Tan H., Papacharalambous J., Valdez H., Mamolo C.Tofacitinib versus etanercept or placebo in patients with moderate to severe chronic plaque psoriasis: patient-reported outcomes from a Phase 3 study. Journal of the European Academy of Dermatology and Venereology (2016) 30:10 (1753-1759). Date of Publication: 1 Oct 2016 |
| 35 | Asahina A., Etoh T., Igarashi A., Imafuku S., Saeki H., Shibasaki Y., Tomochika Y., Toyoizumi S., Nagaoka M., Ohtsuki M.Oral tofacitinib efficacy, safety and tolerability in Japanese patients with moderate to severe plaque psoriasis and psoriatic arthritis: A randomized, double-blind, phase 3 study. Journal of Dermatology (2016) 43:8 (869-880). Date of Publication: 1 Aug 2016 |
| 36 | Clowse M.E.B., Feldman S.R., Isaacs J.D., Kimball A.B., Strand V., Warren R.B., Xibillé D., Chen Y., Frazier D., Geier J., Proulx J., Marren A.Pregnancy Outcomes in the tofacitinib Safety Databases for Rheumatoid Arthritis and Psoriasis. Drug Safety (2016) 39:8 (755-762). Date of Publication: 1 Aug 2016 |
| 37 | Wolk R., Langley R., Cohen A., Foley P., Griffiths C., Lebwohl M., Leonardi C., Winthrop K., Proulx J., Rottinghaus S., Thompson J., Tatulych S., Mallbris L., Swanson R. Safety of tofacitinib, an oral Janus kinase inhibitor: Integrated data analysis from the global chronic plaque psoriasis clinical trials. Journal of the European Academy of Dermatology and Venereology (2016) 30 Supplement 6 (72-73). Date of Publication: 1 Jul 2016 |
| 38 | Strober B., Checchio T., Gupta P., Mandema J., Wolk R., Valdez H., Tan H., Puig L., Krishnaswami S., Tallman A., Kaur M., Ito K.A dose-response model-based meta-analysis to compare tofacitinib to other psoriasis treatments. Journal of the European Academy of Dermatology and Venereology (2016) 30 Supplement 6 (94-95). Date of Publication: 1 Jul 2016 |
| 39 | Lambert J., Strohal R., De La Cruz C., Thaçi D., Bachelez H., Iversen L., Rottinghaus S., Tallman A., Tan H., Berstein G.Predictors of response to tofacitinib or etanercept in a Phase 3 randomised, non-inferiority study in patients with moderate to severe chronic plaque psoriasis. Journal of the European Academy of Dermatology and Venereology (2016) 30 Supplement 6 (78). Date of Publication: 1 Jul 2016 |
| 40 | Winthrop K., Korman N., Abramovits W., Valdez H., Rottinghaus S., Tan H., Gardner A., Mukwaya G., Kaur M.T cell-mediated immune response to pneumococcus and tetanus toxoid vaccines in patients with moderate to severe psoriasis following longterm oral tofacitinib treatment. Journal of the European Academy of Dermatology and Venereology (2016) 30 Supplement 6 (81-82). Date of Publication: 1 Jul 2016 |
| 41 | Kaur M., Merola J., Tatulych S., Mamolo C., Lan S.Efficacy of tofacitinib for the treatment of nail psoriasis: Two 52-week Phase 3 studies in patients with moderate to severe plaque psoriasis. Journal of the European Academy of Dermatology and Venereology (2016) 30 Supplement 6 (81). Date of Publication: 1 Jul 2016 |
| 42 | Busard C., Pasch M., Oudshoorn S., De Vries A.C., Hooft L., Spuls P.Interventions for nail psoriasis. Journal of the European Academy of Dermatology and Venereology (2016) 30 Supplement 6 (100-101). Date of Publication: 1 Jul 2016 |
| 43 | Bing N., Zhou H.Y., Zhang B.H., Nagaoka M., Valdez H., Vincent M., Clark J.D.Genome-wide trans-ancestry meta-analysis of herpes zoster in rheumatoid arthritis and psoriasis patients treated with tofacitinib. Annals of the Rheumatic Diseases (2016) 75 Supplement 2 (256-257). Date of Publication: 1 Jun 2016 |
| 44 | Korman N.J., King B., Wolk R., Tan H., Kaur M., Robertson D., Langley R.Efficacy and safety of oral tofacitinib in North American patients with moderate to severe plaque psoriasis: Pooled analyses of data from randomized phase 3 studies. Journal of the American Academy of Dermatology (2016) 74:5 SUPPL. 1 (AB247). Date of Publication: May 2016 |
| 45 | Papp K.A., Krueger J.G., Feldman S.R., Langley R.G., Thaci D., Torii H., Tyring S., Wolk R., Gardner A., Mebus C., Tan H., Luo Y., Gupta P., Mallbris L., Tatulych S.Tofacitinib, an oral Janus kinase inhibitor, for the treatment of chronic plaque psoriasis: Long-term efficacy and safety results from 2 randomized phase-III studies and 1 open-label long-term extension study. Journal of the American Academy of Dermatology (2016) 74:5 (841-850). Date of Publication: 1 May 2016 |
| 46 | Paul C., Langley R.G., Valenzuela F., Cather J., Gardner A., Proulx J., Tan H., Wolk R., Kaur M., Rottinghaus S.Tofacitinib in patients with moderate to severe chronic plaque psoriasis: 2-year efficacy and safety in an open-label long-term extension study. Journal of the American Academy of Dermatology (2016) 74:5 SUPPL. 1 (AB278). Date of Publication: May 2016 |
| 47 | Ludbrook V.J., Hicks K.J., Hanrott K.E., Patel J.S., Binks M.H., Wyres M.R., Watson J., Wilson P., Simeoni M., Schifano L.A., Reich K., Griffiths C.E.M.Investigation of selective JAK1 inhibitor GSK2586184 for the treatment of psoriasis in a randomized placebo-controlled phase IIa study. British Journal of Dermatology (2016) 174:5 (985-995). Date of Publication: 1 May 2016 |
| 48 | Merola J., Tatulych S., Mamolo C., Lan S., Mallbris L., Kaur M.Efficacy of tofacitinib for the treatment of nail psoriasis: Two 52-week phase 3 studies in patients with moderate to severe plaque psoriasis. Journal of the American Academy of Dermatology (2016) 74:5 SUPPL. 1 (AB248). Date of Publication: May 2016 |
| 49 | Lambert J., Strohal R., De La Cruz C., Thaçi D., Bachelez H., Iversen L., Rottinghaus S., Tallman A., Tan H., Berstein G.Predictors of response to tofacitinib or etanercept in a phase 3 randomized, noninferiority study in patients with moderate to severe chronic plaque psoriasis. Journal of the American Academy of Dermatology (2016) 74:5 SUPPL. 1 (AB263). Date of Publication: May 2016 |
| 50 | Tan H., Valdez H., Wolk R., Tallman A., Gordon K.B.Early clinical response as a predictor of subsequent response to tofacitinib treatment: Results from two phase 3 studies of subjects with moderate to severe plaque psoriasis. Journal of the American Academy of Dermatology (2016) 74:5 SUPPL. 1 (AB244). Date of Publication: May 2016 |
| 51 | Winthrop K., Korman N., Sofen H., Abramovits W., Valdez H., Rottinghaus S., Tan H., Gardner A., Kaur M., Mukwaya G.T cell-mediated immune response to Pneumococcus and tetanus toxoid vaccines in patients with moderate to severe psoriasis following long-term oral tofacitinib treatment. Journal of the American Academy of Dermatology (2016) 74:5 SUPPL. 1 (AB277). Date of Publication: May 2016 |
| 52 | Thaçi D., Griffiths C.E.M., Bundy C., Korman N.J., Lan S., Robertson D., Tatulych S., Tallman A., Kaur M., Mamolo C.Improvements in health-related quality of life and symptoms of depression with tofacitinib: Results from two randomized phase 3 studies in patients with moderate to severe psoriasis. Journal of the American Academy of Dermatology (2016) 74:5 SUPPL. 1 (AB255). Date of Publication: May 2016 |
| 53 | Menter M.A., Papp K.A., Cather J., Leonardi C., Pariser D.M., Krueger J.G., Wohlrab J., Amaya-Guerra M., Kaszuba A., Nadashkevich O., Tsai T.-F., Gupta P., Tan H., Valdez H., Mallbris L., Tatulych S. Efficacy of tofacitinib for the treatment of moderate-to-severe chronic plaque psoriasis in patient subgroups from two randomised phase 3 trials. Journal of Drugs in Dermatology (2016) 15:5 (568-580). Date of Publication: 1 May 2016 |
| 54 | Krueger J., Clark J.D., Suárez-Fariñas M., Fuentes-Duculan J., Cueto I., Wang C.Q., Tan H., Wolk R., Rottinghaus S.T., Whitley M.Z., Valdez H., Von Schack D., O'Neil S.P., Reddy P.S., Tatulych S.Tofacitinib attenuates pathologic immune pathways in patients with psoriasis: A randomized phase 2 study. Journal of Allergy and Clinical Immunology (2016) 137:4 (1079-1090). Date of Publication: 1 Apr 2016 |
| 55 | Hutmacher M.M., Papp K.A., Lebwohl M., Ito K., Tan H., Wolk R., Mebus C., Rottinghaus S., Valdez H., Krishnaswami S., Gupta P.Exposure-response characteristics and predictors of efficacy in moderate to severe chronic plaque psoriasis patients receiving tofacitinib. Clinical Pharmacology and Therapeutics (2016) 99 SUPPL. 1 (S52). Date of Publication: February 2016 |
| 56 | Papp K., Krueger J., Feldman S., Langley R.G.B., Torii H., Tyring S.K., Tatulych S., Gupta P., Mebus C., Tan H., Luo Y., Mamolo C., Mallbris L., Wolk R.Efficacy, safety and patient-reported outcomes up to 52 weeks with tofacitinib, an oral Janus kinase inhibitor, for the treatment of chronic plaque psoriasis: Results from two randomised, Phase 3 trials. Journal of Investigative Dermatology (2015) 135 SUPPL. 3 (S10). Date of Publication: October 2015 |
| 57 | Langley R.G.B., Cohen A.D., Foley P., Griffiths C.E.M., Lebwohl M., Leonardi C., Winthrop K., Proulx J., Rottinghaus S.T., Wolk R., Thompson J.R., Tatulych S., Mallbris L., Swanson R. Safety of tofacitinib, an oral Janus kinase inhibitor, for the treatment of chronic plaque psoriasis: Integrated data analysis from the global clinical trials. Journal of Investigative Dermatology (2015) 135 SUPPL. 3 (S9). Date of Publication: October 2015 |
| 58 | Papp K.A., Menter M.A., Abe M., Elewski B., Feldman S.R., Gottlieb A.B., Langley R., Luger T., Thaci D., Buonanno M., Gupta P., Proulx J., Lan S., Wolk R.Tofacitinib, an oral Janus kinase inhibitor, for the treatment of chronic plaque psoriasis: Results from two randomized, placebo-controlled, phase III trials. British Journal of Dermatology (2015) 173:4 (949-961). Date of Publication: 1 Oct 2015 |
| 59 | Merola J., Tatulych S., Kaur M., Lan S., Mallbris L., Mamolo C.Efficacy of tofacitinib for the treatment of nail psoriasis: Two 52-week phase 3 studies in patients with moderate to severe plaque psoriasis. Journal of Investigative Dermatology (2015) 135 SUPPL. 3 (S9). Date of Publication: October 2015 |
| 60 | Feldman S., Kimball A.B., Warren R.B., Frazier D., Proulx J., Marren A.Pregnancy outcomes in the tofacitinib psoriasis safety database up to April 2014. Journal of Investigative Dermatology (2015) 135 SUPPL. 3 (S9). Date of Publication: October 2015 |
| 61 | Gupta P., Hutmacher M., Papp K., Lebwohl M., Ito K., Tan H., Wolk R., Mebus C., Rottinghaus S.T., Valdez H., Mallbris L., Krishnaswami S.Tofacitinib exposure-response characteristics in patients with moderate to severe chronic plaque psoriasis. Journal of Investigative Dermatology (2015) 135 SUPPL. 3 (S8). Date of Publication: October 2015 |
| 62 | Winthrop K., Lebwohl M., Cohen A.D., Weinberg J., Tyring S.K., Rottinghaus S.T., Gupta P., Ito K., Thompson J.R., Kaur M., Egeberg A., Mallbris L., Valdez H.Herpes zoster and tofacitinib therapy in patients with psoriasis. Journal of Investigative Dermatology (2015) 135 SUPPL. 3 (S10). Date of Publication: October 2015 |
| 63 | Papp K., Menter A., Raman M., Janes J., Disch D., Macias W.A Phase 2b dose-ranging trial of baricitinib, an oral JAK 1/JAK 2 inhibitor, in patients with moderate-to-severe psoriasis: Results from the randomized withdrawal and re-treatment periods. Journal of Investigative Dermatology (2015) 135 SUPPL. 3 (S7). Date of Publication: October 2015 |
| 64 | Papp K., Pariser D., Catlin M., Wierz G., Ball G., Akinlade B., Zeiher B., Krueger J.G.A phase 2a randomized, double-blind, placebo-controlled, sequential dose-escalation study to evaluate the efficacy and safety of ASP015K, a novel Janus kinase inhibitor, in patients with moderate-to-severe psoriasis. British Journal of Dermatology (2015) 173:3 (767-776). Date of Publication: 1 Sep 2015 |
| 65 | Berstein G., Zhang W., Soderstrom C., Fraser S., Lee J., Quazi A., Wolk R., Mebus C., Valdez H., Fitz L.Serum IL-17A at baseline correlates with clinical response to tofacitinib and etanercept in moderate to severe psoriasis. Journal of Investigative Dermatology (2015) 135 SUPPL. 2 (S33). Date of Publication: September 2015 |
| 66 | Seminario-Vidal L., Cantrell W., Elewski B.E.Pulmonary cryptococcosis in the setting of tofacitinib therapy for psoriasis. Journal of Drugs in Dermatology (2015) 14:8 (901-902). Date of Publication: 1 Aug 2015 |
| 67 | Valenzuela F., Papp K.A., Pariser D., Tyring S.K., Wolk R., Buonanno M., Wang J., Tan H., Valdez H.Effects of tofacitinib on lymphocyte sub-populations, CMV and EBV viral load in patients with plaque psoriasis. BMC Dermatology (2015) 15:1 Article Number: 08. Date of Publication: 8 May 2015 |
| 68 | Bissonnette R., Iversen L., Sofen H., Griffiths C.E.M., Foley P., Romiti R., Bachinsky M., Rottinghaus S.T., Tan H., Proulx J., Valdez H., Gupta P., Mallbris L., Wolk R.Tofacitinib withdrawal and retreatment in moderate-to-severe chronic plaque psoriasis: A randomized controlled trial. British Journal of Dermatology (2015) 172:5 (1395-1406). Date of Publication: 1 May 2015 |
| 69 | Papp K.A., Menter M.A., Abe M., Elewski B.E., Feldman S.R., Gottlieb A.G., Thaci D., Luger T., Tatulych S., Gupta P., Proulx J., Lan S.-P., Wolk R.Two phase 3 studies of oral tofacitinib in patients with moderate to severe plaque psoriasis: 16-week efficacy and safety results. Journal of the American Academy of Dermatology (2015) 72:5 SUPPL. 1 (AB66). Date of Publication: May 2015 |
| 70 | Bissonnette R., Iversen L., Sofen H., Griffiths C.E.M., Foley P., Romiti R., Bachinsky M., Rottinghaus S.T., Tan H., Proulx J., Valdez H., Gupta P., Mallbris L., Wolk R.Tofacitinib withdrawal and retreatment in moderate-to-severe chronic plaque psoriasis: A phase 3 randomized trial. Australasian Journal of Dermatology (2015) 56 SUPPL. 2 (37). Date of Publication: May 2015 |
| 71 | Gordon K., Strober B., Tan H., Yang L., Wolk R., Mallbris L., Valdez H.Early clinical response as a predictor of efficacy in moderate to severe psoriasis patients treated with tofacitinib in a phase 2 study. Journal of the American Academy of Dermatology (2015) 72:5 SUPPL. 1 (AB231). Date of Publication: May 2015 |
| 72 | Valenzuela F., Lahfa M., Tan H., Papacharalambous J., Mamolo C.Tofacitinib or etanercept versus placebo on patient-reported outcomes: Results from a Phase 3 study in moderate to severe chronic plaque psoriasis. Journal of Clinical and Aesthetic Dermatology (2015) 8:5 Supplement 1 (S19-S20). Date of Publication: 1 May 2015 |
| 73 | Alinia H., Feldman S.R.Oral tofacitinib for psoriasis: What happens with interrupted treatment?. British Journal of Dermatology (2015) 172:5 (1194-1195). Date of Publication: 1 May 2015 |
| 74 | Mamolo C.M., Bushmakin A.G., Cappelleri J.C.Application of the Itch Severity Score in patients with moderate-to-severe plaque psoriasis: Clinically important difference and responder analyses. Journal of Dermatological Treatment (2015) 26:2 (121-123). Date of Publication: 1 Apr 2015 |
| 75 | Bachelez H., Van De Kerkhof P.C.M., Strohal R., Kubanov A., Valenzuela F., Lee J.-H., Yakusevich V., Chimenti S., Papacharalambous J., Proulx J., Gupta P., Tan H., Tawadrous M., Valdez H., Wolk R.Tofacitinib versus etanercept or placebo in moderate-to-severe chronic plaque psoriasis: A phase 3 randomised non-inferiority trial. The Lancet (2015) 386:9993 (552-561). Date of Publication: 8 Aug 2015 |
| 76 | Bushmakin A.G., Mamolo C., Cappelleri J.C., Stewart M.The relationship between pruritus and the clinical signs of psoriasis in patients receiving tofacitinib. Journal of Dermatological Treatment (2015) 26:1 (19-22). Date of Publication: 1 Feb 2015 |
| 77 | Krueger J., Suarez-Farinas M., Fuentes-Duculan J., Cueto I., Mallbris L., Tatulych S., Clark J., Tan H., Wolk R., Rottinghaus S., Whitley M., Valdez H., Von Schack D., Stewart Z., Zhan Y.Pathological immune pathways in psoriasis are rapidly attenuated by tofacitinib treatment. British Journal of Dermatology (2014) 171:6 (e120-e121). Date of Publication: December 2014 |
| 78 | Yang L., Wang G.Phosphorylation of STAT3 involved in the pathogenesis of dysfunctional regulatory t cells in psoriasis. Journal of Dermatology (2014) 41 SUPPL. 1 (11). Date of Publication: October 2014 |
| 79 | Menter A., Disch D., Clemens J., Janes J., Papp K., Macias W.A phase 2b trial of baricitinib, an oral JAK inhibitor, in patients with moderate to severe psoriasis. Journal of the American Academy of Dermatology (2014) 70:5 SUPPL. 1 (AB162). Date of Publication: May 2014 |
| 80 | Mamolo C., Harness J., Tan H., Menter A.Tofacitinib (CP-690, 550), an oral Janus kinase inhibitor, improves patient-reported outcomes in a phase 2b, randomized, double-blind, placebo-controlled study in patients with moderate-to-severe psoriasisJournal of the European Academy of Dermatology and Venereology (2014) 28:2 (192-203). Date of Publication: February 2014 |
| 81 | Menter A., Papp K.A., Tan H., Tyring S., Wolk R., Buonanno M.Efficacy of tofacitinib, an oral janus kinase inhibitor, on clinical signs of moderate-to-severe plaque psoriasis in different body regions. Journal of Drugs in Dermatology (2014) 13:3 (252-256). Date of Publication: March 2014 |
| 82 | Craiglow B.G., King B.A.Killing two birds with one stone: Oral tofacitinib reverses alopecia universalis in a patient with plaque psoriasis. Journal of Investigative Dermatology (2014) 134:12 (2988-2990). Date of Publication: 13 Dec 2014 |
| 83 | Sandoval L.F., Pierce A., Feldman S.R.Systemic therapies for psoriasis: An evidence-based update. American Journal of Clinical Dermatology (2014) 15:3 (165-180). Date of Publication: July 2014 |
| 84 | Strober B., Buonanno M., Clark J.D., Kawabata T., Tan H., Wolk R., Valdez H., Langley R.G., Harness J., Menter A., Papp K.Effect of tofacitinib, a Janus kinase inhibitor, on haematological parameters during 12 weeks of psoriasis treatment. British Journal of Dermatology (2013) 169:5 (992-999). Date of Publication: November 2013 |
| 85 | Bissonnette R., Foley P., Griffiths C.E.M., Iversen L., Kadurina M., Sofen H.L., Takahashi M.D.F., Bachinsky M.E., Gupta P., Mallbris L., Proulx J., Tan H., Valdez H., Wolk R.Tofacitinib for moderate to severe chronic plaque psoriasis: 24-week preliminary analysis from the 56-week phase 3 opt re-treatment study. Journal of the European Academy of Dermatology and Venereology (2013) 27 SUPPL. 4 (23). Date of Publication: July 2013 |
| 86 | Ports W., Bissonnette R., Papp K., Khan S., Lan S., Mamolo C., Masek-Hammerman K., Purohit V.Tofacitinib ointment efficacy and biomarker improvement in psoriasis. Journal of Investigative Dermatology (2013) 133 SUPPL. 1 (S164). Date of Publication: May 2013 |
| 87 | Tan H., Gupta P., Harness J., Wolk R., Chapel S., Menter A., Strober B., Langley R.G., Krishnaswami S., Papp K.A.Dose response and pharmacokinetics of tofacitinib (CP-690, 550), an oral janus kinase inhibitor, in the treatment of chronic plaque psoriasis. CPT: Pharmacometrics and Systems Pharmacology (2013) 2:5 Article Number: e44. Date of Publication: May 2013 |
| 88 | Valenzuela F., Papp K.A., Pariser D., Tyring S., Wolk R., Buonanno M., Valdez H.Tofacitinib has no clinically significant effects on cells controlling chronic viral infection and reactivation. Journal of Investigative Dermatology (2013) 133 SUPPL. 1 (S17). Date of Publication: May 2013 |
| 89 | Ports W., Bolduc C., Papp K., Lamba M., Bissonnette R., Khan S., Lan S.Efficacy and safety of topical janus kinase inhibitor tofacitinib in the treatment of chronic plaque psoriasis: Results of a phase IIA randomized clinical trial. Journal of the American Academy of Dermatology (2013) 68:4 SUPPL. 1 (AB199). Date of Publication: April 2013 |
| 90 | Gao X., Hutmacher M., Khan S., Lan S., Ports W.Longitudinal PK PD modeling to project the effect sizes and uncertainties of the topical application of tofacitinib in psoriasis patients. Clinical Pharmacology and Therapeutics (2013) 93 SUPPL. 1 (S18). Date of Publication: February 2013 |
| 91 | Ports W.C., Khan S., Lan S., Lamba M., Bolduc C., Bissonnette R., Papp K.A randomized phase 2a efficacy and safety trial of the topical Janus kinase inhibitor tofacitinib in the treatment of chronic plaque psoriasis. British Journal of Dermatology (2013) 169:1 (137-145). Date of Publication: July 2013 |
| 92 | Punwani N., Scherle P., Flores R., Shi J., Liang J., Yeleswaram S., Levy R., Williams W., Gottlieb A.Preliminary clinical activity of a topical JAK1/2 inhibitor in the treatment of psoriasis. Journal of the American Academy of Dermatology (2012) 67:4 (658-664). Date of Publication: October 2012 |
| 93 | Papp K.A., Menter A., Strober B., Langley R.G., Buonanno M., Wolk R., Gupta P., Krishnaswami S., Tan H., Harness J.A.Efficacy and safety of tofacitinib, an oral Janus kinase inhibitor, in the treatment of psoriasis: A Phase 2b randomized placebo-controlled dose-ranging study. British Journal of Dermatology (2012) 167:3 (668-677). Date of Publication: September 2012 |
| 94 | Papp K., Pariser D., Wierz G., Ball G., Akinlade B., Zeiher B.Phase IIa randomized, double-blind, placebocontrolled, sequential dose-escalation study to evaluate the efficacy and safety of ASP015K, a novel Janus kinase (JAK) inhibitor, in patients with moderate-to-severe psoriasis. Dermatology and Therapy (2012) 2 SUPPL. 1 (S34-S35). Date of Publication: July 2012 |
| 95 | Menon S., Boy M.G., Wang C., Wilkinson B.E., Zwillich S.H., Chan G., Krishnaswami S.Single and multiple-dose pharmacokinetics of tofacitinib (CP-690,550) from a double-blind, placebo-controlled, dose-escalation study in medically stable subjects with psoriasis. Clinical Pharmacology and Therapeutics (2012) 91 SUPPL. 1 (S33). Date of Publication: March 2012 |
| 96 | Gupta P., Krishnaswami S., Harness J.Development and application of a model-based decision criterion for a laboratory endpoint to facilitate Tofacitinib (CP-690, 550) Phase 3 dose selection. Clinical Pharmacology and Therapeutics (2012) 91 SUPPL. 1 (S94). Date of Publication: March 2012 |
| 97 | Salcedo C., Michelena P., Ferran M., M Pujol R., Merlos M., Santamaria-Babi L.F.Pharmacological activity of JAK-kinase inhibitors on gene expression of psoriatic skin explants. Journal of Investigative Dermatology (2011) 131 SUPPL. 2 (S20). Date of Publication: September 2011 |
| 98 | Mamolo C.M., Bushmakin A.G., Cappelleri J.C., Stewart M.The effect of oral cp-690, 550 on pruritus in patients with moderate-to-severe plaque psoriasis. Value in Health (2011) 14:3 (A56). Date of Publication: May 2011 |
| 99 | Boy M.G., Wang C., Wilkinson B.E., Chow V.F.-S., Clucas A.T., Krueger J.G., Gaweco A.S., Zwillich S.H., Changelian P.S., Chan G.Double-blind, placebo-controlled, dose-escalation study to evaluate the pharmacologic effect of CP-690, 550 in patients with psoriasis. Journal of Investigative Dermatology (2009) 129:9 (2299-2302). Date of Publication: September 2009 |
| 100 | Soriano E.R., Madariaga H.J., Castañeda O., Citera G., Schneeberger E.E., Cardiel M.H., Hendrikx T., Graham D., Shi H. Liver enzyme abnormalities after tofacitinib treatment in patients with hepatic steatosis from the rheumatoid arthritis, psoriatic arthritis, and psoriasis clinical programs. Journal of Clinical Rheumatology (2019) 25:3 Supplement (S40). Date of Publication: 1 Apr 2019 |
| 101 | Konrad R.J., Higgs R.E., Rodgers G.H., Ming W., Qian Y.-W., Bivi N., Mack J.K., Siegel R.W., Nickoloff B.J. Assessment and Clinical Relevance of Serum IL-19 Levels in Psoriasis and Atopic Dermatitis Using a Sensitive and Specific Novel Immunoassay. Scientific reports (2019) 9:1 (5211). Date of Publication: 26 Mar 2019 |
| 102 | Lloyd-Lavery A. Long-term safety data for tofacitinib, an oral Janus kinase inhibitor, for the treatment for psoriasis. British Journal of Dermatology (2018) 179:4 (815-816). Date of Publication: 1 Oct 2018 |
| 103 | Soriano E.R., Madariaga H., Castañeda O., Citera G., Schneeberger E.E., Cardiel M.H., Hendrikx T., Graham D., Shi H., De Leon D.P. Liver enzyme abnormalities after tofacitinib treatment in patients with hepatic steatosis from the rheumatoid arthritis, psoriatic arthritis, and psoriasis clinical programs. Arthritis and Rheumatology (2018) 70 Supplement 9 (2707-2709). Date of Publication: 1 Sep 2018 |
| 104 | Juliane F., Markus S.Immediate response of therapy-refractory palmoplantar pustular psoriasis and psoriasis arthritis to tofacitinib. Swiss Medical Weekly (2016) 146 Supplement 217 (7S). Date of Publication: 1 Aug 2016 |
| 105 | Bissonnette R., Luchi M., Fidelus-Gort R., Jackson S., Zhang H., Flores R., Newton R., Scherle P., Yeleswaram S., Chen X., Menter A randomized, double-blind, placebo-controlled, dose-escalation study of the safety and efficacy of INCB039110, an oral janus kinase 1 inhibitor, in patients with stable, chronic plaque psoriasis. Journal of Dermatological Treatment 27:4 (332-338) |
| 106 | Bushmakin A.G., Mamolo C., Cappelleri J.C., Stewart M.The relationship between pruritus and the clinical signs of psoriasis in patients receiving tofacitinib. Journal of Dermatological Treatment (2015). Journal of Dermatological Treatment 26:1 (19-22) |
| 107 | Papp K, Gordon K, Thaçi D, Morita A, Gooderham M, Foley P, Girgis IG, Kundu S, Banerjee S.Phase 2 Trial of Selective Tyrosine Kinase 2 Inhibition in Psoriasis. N Engl J Med. 2018 Oct 4;379(14):1313-1321. doi: 10.1056/NEJMoa1806382. Epub 2018 Sep 11. |
| 108 | Banfield C, Scaramozza M, Zhang W, Kieras E, Page KM, Fensome A, Vincent M, Dowty ME, Goteti K, Winkle PJ, Peeva E.The Safety, Tolerability, Pharmacokinetics, and Pharmacodynamics of a TYK2/JAK1 Inhibitor (PF-06700841) in Healthy Subjects and Patients With Plaque Psoriasis. J Clin Pharmacol. 2018 Apr;58(4):434-447. doi: 10.1002/jcph.1046. Epub 2017 Dec 21. |
| 109 | Strober B, Checchio T, Gupta P, Mandema J, Wolk R, Valdez H, Tan H, Puig L, Krishnaswami S, Tallman A, Kaur M, Ito K.A dose-response model-based meta-analysis to compare tofacitinib to other psoriasis treatments. JEADV 2016, 30 (Suppl. 6), 3–105 |
| 110 | Mamolo C, Bissonnette R, Khan S, Lan S, Ports W, Poulin Y. An evaluation of the effect of topical tofacitinib (CP-690, 550) on pruritus and patient satisfaction with study medication in a phase IIA trial for plaque psoriasis. Journal of the American Academy of Dermatology (2013), 68(4) AB192 |
| 111 | Harness J, Menter A, Tan H, Papp K.Tasocitinib (CP-690, 550), an oral Janus kinase inhibitor, significantly improves the symptoms of moderate to severe psoriasis with early onset of efficacy. Journal of the American Academy of Dermatology (2011), 64(2) AB149 |
| 112 | Mamolo C, Bushmakin A, Harness J, Cappelleri J, Stewart M.The itch severity item: Mediation modeling and measurement characteristics from a phase IIb trial of tasocitinib (CP-690, 550), an oral Janus kinase inhibitor, in patients with plaque psoriasis. Journal of the American Academy of Dermatology (2011), 64(2) AB152 |
| 113 | Mamolo C, Bushmakin A, Harness J, Cappelleri J, Stewart M. An evaluation of the effect of tasocitinib (CP-690, 550), an oral Janus kinase inhibitor, on pruritus in patients with plaque psoriasis. Journal of the American Academy of Dermatology (2011), 64(2) AB152 |
| 114 | Papp K, Menter A, Tan H, Harness J.Tasocitinib (CP-690, 550), an oral Janus kinase inhibitor, consistently improves the clinical signs of moderate to severe psoriasis in different body regions. Journal of the American Academy of Dermatology (2011), 64(2) AB153 |
| 115 | Callis Duffin K, Luchi M, Fidelus-Gort R, et al. Novel mechanism for topical treatment of plaque psoriasis – results of a randomized, double blind, concentration ranging, vehicle controlled 12 week study with JAK 1/2 inhibitor INCB018424 cream. The 70th SID meeting; Atlanta, GA, USA. 2010. p. 261. J. Invest. Dermatol. Abstract. |
| 116 | Punwani N, Gottlieb A, Birnbaum J, Williams W.Efficacy and safety of topical INCB018424, a selective Janus kinase 1 & 2 (JAK1&2) inhibitor in psoriasis. Journal of the American Academy of Dermatology (2009), 60(3) AB176 |
| 117 | Improvement in psoriatic lesions during a 14-day trial of CP-690, 550 (CP), an orally active inhibitor of janus kinase 3 (jak3) |

**Table S6. List of excluded studies and criteria for exclusion.**

| **Article** | **Exclusion criteria** |
| --- | --- |
| Liver enzyme abnormalities after tofacitinib treatment in patients with hepatic steatosis from the rheumatoid arthritis, psoriatic arthritis and psoriasis clinical programmes. Soriano E.R., Madariaga H., Castañeda O., Citera G., Schneeberger E.E., Cardiel M.H., Hendrikx T., Graham D., Shi H., Ponce De Leon D. Annals of the Rheumatic Diseases (2018) 77 Supplement 2 (593-594). Date of Publication: 1 Jun 2018 | No scientific review |
| Incidence of thromboembolic events in the tofacitinib rheumatoid arthritis, psoriasis, psoriatic arthritis and ulcerative colitis development programmes. Mease P.J., Kremer J., Cohen S., Curtis J.R., Charles-Schoeman C., Loftus E.V., Greenberg J., Palmetto N., Kanik K.S., Graham D., Wang C., Biswas P., Chan G., DeMasi R., Valdez H., Hendrikx T., Jones T.V. Annals of the Rheumatic Diseases (2018) 77 Supplement 2 (983). Date of Publication: 1 Jun 2018 | No scientific review |
| Strober B.E., Gottlieb A.B., van de Kerkhof P.C.M., Puig L., Bachelez H., Chouela E., Imafuku S., Thaçi D., Tan H., Valdez H., Gupta P., Kaur M., Frajzyngier V., Wolk R.Benefit–risk profile of tofacitinib in patients with moderate-to-severe chronic plaque psoriasis: pooled analysis across six clinical trials. British Journal of Dermatology (2019) 180:1 (67-75). Date of Publication: 1 Jan 2019 | No scientific Review |
| Safety signal detection and evaluation in clinical development programs: A case study of tofacitinib. Dasic G., Jones T., Frajzyngier V., Rojo R., Madsen A., Valdez H. Pharmacology Research and Perspectives (2018) 6:1 Article Number: e00371. Date of Publication: 1 Feb 2018. | No scientific review |
| Efficacy of Systemic Treatments of Psoriasis on Pruritus: A ID Systemic Literature Review and Meta-Analysis. Théréné C., Brenaut E., Barnetche T., Misery L. Journal of Investigative Dermatology (2018) 138:1 (38-45). Date of Publication: 1 Jan 2018 | Pruritus |
| Validation of the itch severity item as a measurement tool for pruritus in patients with psoriasis: Results from a phase 3 tofacitinib program. Ständer S., Luger T.A., Cappelleri J.C., Bushmakin A.G., Mamolo C., Zielinski M.A., Tallman A.M., Yosipovitch G. Acta Dermato-Venereologica (2018) 98:3 (340-345). Date of Publication: 2018 | No scientific review |
| Benefit–risk profile of tofacitinib in patients with moderate-to-severe chronic plaque psoriasis: pooled analysis across six clinical trials. Strober B.E., Gottlieb A.B., van de Kerkhof P.C.M., Puig L., Bachelez H., Chouela E., Imafuku S., Thaçi D., Tan H., Valdez H., Gupta P., Kaur M., Frajzyngier V., Wolk R. British Journal of Dermatology (2018). Date of Publication: 2018 | No scientific review |
| **A**dverse drug events associated with 5mg versus 10mg tofacitinib (Janus kinase inhibitor) twice daily for the treatment of autoimmune diseases: A systematic review and meta-analysis of randomized controlled trials. Huang F., Luo Z.-C. Clinical Rheumatology (2018). Date of Publication: 2018 | No scientific review |
| Malik S.U., Muzaffar K., Bilal J., Faridi W., Muddassir S. Serious infections among psoriatic arthritis patients taking TNF inhibitors versus non-TNF biologics: A systematic review and network meta-analysis. Arthritis and Rheumatology (2018) 70 Supplement 9 (1815-1816). Date of Publication: 1 Sep 2018 | psoriatic arthritis |
| Quantitative Evaluations of Time-Course and Treatment Effects of Systemic Agents for Psoriasis: A Model-Based Meta-Analysis. Checchio T., Ahadieh S., Gupta P., Mandema J., Puig L., Wolk R., Valdez H., Tan H., Krishnaswami S., Tallman A., Kaur M., Ito K. Clinical Pharmacology and Therapeutics (2017) 102:6 (1006-1016). Date of Publication: 1 Dec 2017 | Non scientific  review |
| Tofacitinib for the treatment of moderate to severe chronic plaque psoriasis in Japanese patients: Subgroup analyses from a randomized, placebo-controlled phase 3 trial. Abe M., Nishigori C., Torii H., Ihn H., Ito K., Nagaoka M., Isogawa N., Kawaguchi I., Tomochika Y., Kobayashi M., Tallman A.M., Papp K.A. Journal of Dermatology (2017) 44:11 (1228-1237). Date of Publication: 1 Nov 2017 | No scientific review |
| Hashimoto T., Sakai K., Sanders K.M., Yosipovitch G., Akiyama T. Antipruritic effects of janus kinase inhibitor tofacitinib in a mouse model of psoriasis. Acta Dermato-Venereologica (2019) 99:3 (298-303). Date of Publication: 2019 | Non human |
| Effect of tofacitinib on efficacy and patient-reported outcomes in psoriasis patients with baseline psoriatic arthritis: A pooled analysis of 2 phase 3 studies. Bachelez H., Griffiths C.E.M., Papp K., Hall S., Merola J.F., Feldman S.R., Khraishi M., Tallman A., Tan H., Hsu M.-A. Arthritis and Rheumatology (2017) 69 Supplement 10. Date of Publication: 1 Oct 2017 | Non scientific review |

**0Table S7. Metadata article-references**

| **ID paper** | **Treatment**  **Systemic/**  **topical** | **Publication**  **Type/ Journal medical**  **speciality** | **National-multinational**  **Uni/**  **multicentric** | **Total article/**  **Dermatology /Pharma/**  **Research inst/**  **authors** | **Interest conflict** | **Funding** |
| --- | --- | --- | --- | --- | --- | --- |
| 1 | Tofacitinib  systemic | Full text  dermatology | Multinational-EEUU  Multicentric(9) | 11,3,6,0  Pharma | 11 | Pfizer |
| 2 | Tofacitinib  Systemic | Full text  dermatology | EEUU  Multicentric(5) | 10,0,6,0  Pharma | 10 | Pfizer |
| 3 | Tofacitinib  systemic | Full text  pharmacology | Multinational-  EEUU  Multicentric(7) | 11,0,9,2  Pharma | 11 | Pfizer |
| 4 | Abrocitinib systemic | Full text  dermatology | EEUU  Multicentric 4) | 12,1,9,2  Pharma | 12 | Pfizer |
| 5 | Tofacitinib  systemic | Full text  dermatology | EEUU  Multicentric(7) | 9,1,6,2  Pharma | 9 | Pfizer |
| 6 | Tofacitinib  systemic | Abstract congress  rheumatology | Multinational-  Argentina  Multicentric(8) | 10,0,3,5  research inst | 6 | Pfizer |
| 7 | Tofacitinib  systemic | Abstract  Congress  rheumatology | EEUU  Multicentric  (10) | 17,0,10,7  Pharma | 17 | Pfizer |
| 8 | Tofacitinib  systemic | Full text  dermatology | Taiwan  Multicentric(3) | 4,1,0,3  research inst | 2 | Chang Gung Memorial Hospital, Chiayi CMRPG6D0353 |
| 9 | Tofacitinib  systemic | Full text  pharmacology | EEUU  Multicentric (3) | 6,0,6,0  Pharma | 3 | Pfizer |
| 10 | Tofacitinib  systemic | Full text  dermatology | EEUU  Multicentric (6) | 11,5,6,0  Dermatology | 10 | National Psoriasis Foundation Discovery Grant |
| 11 | Tofacitinib  systemic | Full text  dermatology | France  Multicentric (2) | 4,0,2,1  Dermatology | 2 | NR |
| 12 | Tofacitinib  systemic | Full text  dermatology | Multinational-germany  Multicentric (5) | 8,3,5,0  Pharma | 2 | Pfizer |
| 13 | Tofacitinib  systemic | Full text  dermatology | Multinational-EEUU  Multicentric  (10) | 14,3,6,3  Pharma | 12 | Pfizer |
| 14 | Tofacitinib  systemic | Full text  rheumatology | China  unicentric | 2,0,0,0  Internal medicine | 0 | National Natural Science Foundation of China (No. 81560046, 81760057) and Guangxi Natural Science Foundation (No. 2016GXNSFAE380002). |
| 15 | Tofacitinib  systemic | Full text  dermatology | Multinational-  France  Multicentric (8) | 12,5,0,6  Dermatology | 1 | NR |
| 16 | Tofacitinib  systemic | Full text  pharmacology | Multinational-EEUU  Multicentric (5) | 12,1,9,1  Pharma | 12 | Pfizer |
| 17 | Tofacitinib  systemic | Full text  dermatology | Multinational-  Japan  Multicentric (8) | 12,2,6,3  Pharma | 8 | Pfizer |
| 18 | Tofacitinib  Ruxolitinib  systemic | Abstact  Congress  dermatology | EEUU  Multicentric (2) | 5,0,0,0  Research insterest inst | NR | NR |
| 19 | Tofacitinib  systemic | Full text  dermatology | Multinational-  China  Multicentric (15) | 15,11,4,0  Dermatology | 15 | Pfizer |
| 20 | Tofacitinib  systemic | Abstract  Congress  rheumatology | Multinational-  France  Multicentric(9) | 10,1,3,6  Research insterest | 10 | NR |
| 21 | Tofacitinib  systemic | Full text  dermatology | China  Multicentric (2) | 6/6/0/0  Dermatology | 0 | National Natural Science Foundation of China |
| 22 | Tofacitinib  systemic | Full text  Internal medicine | Multinational- EEUU  Multicentric (6) | 8/1/5/2  Pharma | 8 | Pfizer |
| 23 | Tofacitinib  systemic | Full Text  dermatology | France  Unicentric | 4/4/0/0  Dermatology | NR | NR |
| 24 | Tofacitinib  systemic | Full Text  dermatology | Multinational  EEUU  Multicentric (13) | 13/2/6/3  Pharma | 12 | Pfizer |
| 25 | Tofacitinib  systemic | Full text  dermatology | EEUU  Multicentrict (6) | 6/0/4/2  pharma | 6 | Pfizer |
| 26 | Tofacitinib  systemic | Full text  pharmacology | Multionational  Canada  Multicentric (3) | 10/0/7/3  Pharma | 10 | Pfizer |
| 27 | Tofacitinib  systemic | Full text  dermatology | Germany  unicentric | 4/3/0/1  Dermatology | 0 | NR |
| 28 | Tofacitinib  systemic | Full text dermatology | Multinational  EEUU  Multicentric (8) | 9/1/5/2  Pharma | 9 | Pfizer |
| 29 | Tofacitinib  systemic | Full text  dermatology | Taiwan  Multicentric (2) | 2/2/0/0  Dermatology | 2 | NR |
| 30 | Tofacitinib  systemic | Full text  dermatology | Multinational  EEUU  Multicentric (5) | 7/3/4/0  Pharma | 7 | Pfizer |
| 31 | Tofacitinib  systemic | Full text  dermatology | Multinational  EEUU  Multicentric (9) | 12/2/7/3  Pharma | 12 | Pfizer |
| 32 | Tofacitinib  systemic | Full text  dermatology | Multinational  EEUU  Multicentric (9) | 10/3/4/3  Pharma | 10 | Pfizer |
| 33 | Tofacitinib  systemic | Full text  dermatoogy | Multinational  EEUU  Multicentric (9) | 11/3/4/4  pharma-research Inst | 11 | Pfizer |
| 34 | Tofacitinib  systemic | Full text  dermatology | Multinational  EEUU  Multicentric (5) | 7/1/5/1  pharma | 7 | Pfizer |
| 35 | Tofacitinib  systemic | Full text  dermatology | Japan  Multicentric (7) | 10/6/4/0  Dermatology | 9 | Pfizer |
| 36 | Tofacitinib  systemic | Full text  dermatology | Multinational  EEUU  Multicentric (11) | 12/3/5/3  Pharma | 12 | Pfizer |
| 37 | Tofacitinib  systemic | Abstract congress  dermatolgy | Multinaitonal  EEUU  Multicentric (11) | 14/3/7/7  Pharma  Research inst | 14 | Pfizer |
| 38 | Tofacitinib  systemic | Abstract congress  dermatology | Multinational  EEUU  Multicentric (9) | 12/2/10/0  Pharma | 12 | NR |
| 39 | Tofacitinib  systemic | Abstract congress  dermatology | Multinational  EEUU  Multicentric (9) | 10/3/3/4  University | NR | Pfizer |
| 40 | Tofacitinib  systemic | Abstract congress  dermatology | EEUU  Multicentric (9) | 9/1/6/2  Pharma | 9 | NR |
| 41 | Tofacitinib  systemic | Abstract  Congress  dermatology | EEUU  Multicentric (3) | 5/1/3/1  Pharma | 5 | NR |
| 42 | Tofacitinib  systemic | Abstract  Congress  dermatology | Netherlands  Multicentric (3) | 6/5/0/1  Dermatology | 2 | NR |
| 43 | Tofacitinib  systemic | Abstract  Congress  rheumatology | Multinational  EEUU  Multicentric (4) | 8/0/8/0  Pharma | 8 | Pfizer |
| 44 | Tofacitinib  systemic | Abstract  Congress  dermatology | Multinational-EEUU  Multicentric (4) | 8/0/5/3  Pharma | NR | Pfizer |
| 45 | Tofacitinib  systemic | Full text  dermatology | Multinational-  Canada  Multicentric (10) | 16/3/7/6  Pharma | 16 | Pfizer |
| 46 | Tofacitinib  systemic | Abstract  Congress  dermatology | Multinational-  EEUU  Multicentric (7) | 10/1/6/3  Pharma | NR | Pfizer |
| 47 | solcitinib (GSK2586184)  systemic | Full text  dermatology | Multinational-  EEUU  Multicentric (5) | 12/2/10/0  Pharma | 12 | GlaxoSmithKline |
| 48 | Tofacitinib  systemic | Abstract  Congress  dermatology | EEUU  Multicentric (3) | 6/0/5/1/0  Pharma | NR | Pfizer |
| 49 | Tofacitinib  systemic | Abstract  Congress  dermatology | Multinational-  EEUU  Multicentric (9) | 10/2/4/4  Pharma  Research insterest | NR | Pfizer |
| 50 | Tofacitinib  systemic | Abstract  Congress  dermatology | EEUU  Multicentric (3) | 5/0/4/1  Pharma | NR | Pfizer |
| 51 | Tofacitinib  systemic | Abstract  Congress  dermatology | EEUU  Multicentric (8) | 10/1/6/3  Pharma | NR | Pfizer |
| 52 | Tofacitinib  systemic | Abstract  Congress  dermatology | Multinational-  Germany  Multicentric (6) | 10/0/6/4  Pharma | NR | Pfizer |
| 53 | Tofacitinib  systemic | Full text  dermatology | Multinational-  EEUU  Multicentric (14) | 16/2/5/9  Research insterest |  |  |
| 54 | Tofacitinib  systemic | Full text  dermatology | EEUU  Multicentric (4) | 15/0/10/5  Pharma | 11 | Pfizer |
| 55 | Tofacitinib  systemic | Abstract congress  Pharmacology | Multinational-  EEUU  Multicentric (5) | 11/1/8/2  Pharma | NR | NR |
| 56 | Tofacitinib  systemic | Abstract congress  dermatology | Multinational-  Canada  Multicentric (8) | 15/0/8/7  Pharma | 15 | NR |
| 57 | Tofacitinib  systemic | Abstract congress  dermatology | Multinational-  Canada  Multientric (10) | 14/0/7/7  Pharma Research insterest | 14 | NR |
| 58 | Tofacitinib  systemic | Full text  dermatology | Multinational-Canada  Multicentric (10) | 14/0/5/9  Research insterest | 14 | pfizer |
| 59 | Tofacitinib  systemic | Abstract congress  dermatology | EEUU  Multicentric (3) | 6/0/5/1  Pharma | 6 | NR |
| 60 | Tofacitinib  systemic | Abstract congress  dermatology | Multinational-  Canada  Multicentric (5) | 6/0/3/3  Pharma  Research insterest | 5 | NR |
| 61 | Tofacitinib  systemic | Abstract  Congress  dermatology | Multinational-  EEUU  Multicentric (5) | 12/0/8/4  Pharma | 12 | NR |
| 62 | Tofacitinib  systemic | Abstract  Congress  dermatology | EEUU  Multicentric (9) | 13/0/8/5  Pharma | 13 | NR |
| 63 | Baricitinib  systemic | Full text  dermatology | Multinational-  EEUU  Multicentric (3) | 6/1/3/2  Pharma | 6 | NR |
| 64 | peficitinib (ASP015K)  systemic | Full text  dermatology | Canada  Multicentric (4) | 8/0/4/4  Pharma | 8 | Astellas |
| 65 | Tofacitinib  systemic | Abstract  Congress  dermatology | EEUU  Multicentric (3) | 10/0/10/0  Pharma | NR | NR |
| 66 | Tofacitinib  systemic | Letter  dermatology | EEUU  Unicentric | 3/3/0/0  Dermatology |  |  |
| 67 | Tofacitinib  systemic | Full text  dermatology | Multinational-  EEUU  Multicentric (4) | 9/3/6/0  Pharma | 9 | Pfizer |
| 68 | Tofacitinib  systemic | Full text  dermatology | Multinational-  EEUU  Multicentric (9) | 14/1/8/5  Pharma | 14 | Pfizer |
| 69 | Tofacitinib  systemic | Abstract  Congress  dermatology | Multinational-  EEUU  Multicentric (8) | 13/1/5/7  University | NR | NR |
| 70 | Tofacitinib  systemic | Abstract  Congress  dermatolofy | Multinational-  EEUU  Multicentric (9) | 14/3/8/3  Pharma | NR | NR |
| 71 | Tofacitinib  systemic | Abstract  Congress  dermatolofy | EEUU  Multicentric (4) | 7/0/5/2  Pharma | NR | Pfizer |
| 72 | Tofacitinib  systemic | Abstract  Congress  dermatology | Multinational-  EEUU  Multicentric (3) | 5/0/3/2  Pharma |  |  |
| 73 | Tofacitinib  systemic | Letter  dermatology | EEUU  Multicentric (2) | 2/2/0/0  Dermatology | 2 | Galderma Laboratories, L.P |
| 74 | Tofacitinib  systemic | Full text  dermatology | EEUU  unicentric | 3/0/3/0  Pharma | 3 | Pfizer |
| 75 | Tofacitinib  systemic | Full text  dermatology | Multinational-  EEUU  Multicentric (9) | 15/4/7/4  Pharma | 15 | Pfizer |
| 76 | Tofacitinib  systemic | Full text  dermatology | EEUU  unicentric | 4/0/4/0  Pharma | 4 | Pfizer |
| 77 | Tofacitinib  systemic | Abstract congress  dermatology | Multinational-  EEUU  Multicentric (5) | 15/0/11/4  Pharma | 4 | Pfizer |
| 78 | Tofacitinib  systemic | Abstract  Congress  dermatology | China  unicentric | 2/0/2/0  Dermatology | NR | NR |
| 79 | Baricitinib  systemic | Abstract  Congress  dermatology | Multinational-  EEUU  Multicentric (3) | 6/0/4/2  Pharma | NR | Eli Lilly |
| 80 | Tofacitinib  systemic | Full text  dermatology | Multinational-  EEUU  Multicentric (3) | 5/0/1/4  Pharma | 4 | Pfizer |
| 81 | Tofacitinib  systemic | Full text  dermatology | Multinational-  EEUU  Multicentric (4) | 6/1/3/2  Pharma |  |  |
| 82 | Tofacitinib  systemic | Full text  dermatology | EEUU  Unicentric | 2/2/0/0  Dermatology | NR | NR |
| 83 | Tofacitinib  systemic | Full text  dermatology | EEUU  Multicentric (3) | 3/3/0/0  Dermatology | 1 | NR |
| 84 | Tofacitinib  systemic | Full text  dermatology | Multinational-  EEUU  Multicentric (8) | 11/2/7/2  Pharma | 11 | Pfizer |
| 85 | Tofacitinib  systemic | Abstract  Congress  dermatology | Multinational-  EEUU  Multicentric (8) | 14/2/7/5  Pharma | 13 | NR |
| 86 | Tofacitinib  topical | Abstract  Congress  dermatology | Multinational-  EEUU  Multicentric (4) | 8/0/5/3  Pharma | NR | NR |
| 87 | Tofacitinib  systemic | Full text  dermatology | Multinational-  EEUU  Multicentric (7) | 10/0/5/5  Pharma | 10 | Pfizer |
| 88 | Tofacitinib  systemic | Abstract  Congress  dermatology | Multinational-  EEUU  Multicentric (5) | 7/0/4/3  Pharma | NR | NR |
| 89 | Tofacitinib  topical | Abstract  Congress  dermatolofy | Multinational-  EEUU  Multicentric (4) | 7/0/4/3  Pharma | NR | Pfizer |
| 90 | Tofacitinib  topical | Abstract congress  dermatology | EEUU  Multicentric (3) | 5/0/4/1  Pharma | 5 | Pfizer |
| 91 | Tofacitinib  topical | Full tex  dermatology | Multinational-  EEUU  Multicentric (5) | 7/0/4/3  Pharma | 5 | Pfizer |
| 92 | Ruxolitinb  topical | Full texy  dermatology | EEUU  Multicentric (2) | 9/1/8/0  Pharma | 9 | Incyte Corp |
| 93 | Tofacitinib  systemic | Full text  dermatology | Multinational-EEUU  Multicentric (5) | 10/0/6/4  Pharma | 10 | Pfizer |
| 94 | ASP015 K  systemic | Abstrac  Congress  dermatology | Multinational-  EEUU  Multicentric (3) | 6/0/4/2  Pharma | NR | NR |
| 95 | Tofacitinib  systemic | Abstrac  Congress  dermatology | EEUU  Unicentric | 7/0/7/0  Pharma | 7 | Pfizer |
| 96 | Tofacitinib  systemic | Abstrac  Congress  dermatology | EEUU  Multicentric (2) | 3/0/3/0  Pharma | 3 | Prizer |
| 97 | Tofacitinib  Systemic  /topical | Abstrac  Congress  dermatology | Spain  Multicentric (3) | 6/3/3/0  Pharma-  dermatology | NR | NR |
| 98 | Tofacitinib  systemic | Abstrac  Congress  dermatology | EEUU  Multicentric (2) | 4/0/4/0  pharma | 4 | Pfizer |
| 99 | Tofacitinib  systemic | Letter  dermatology | EEUU  Multicentric (4) | 11/0/9/2  Pharma | 10 | Pfizer |
| 100 | Tofacitinib  systemic | Abstract  Congress  rheumatology | Multinational-  Argentima  Multicentric (7) | 10/0/4/6  Research insterest | NR | NR |
| 101 | Baricitinib  systemic | Full text  general | EEUU  Multicentric (2) | 9/0/9/0  Pharma | 9 | Eli Lilly |
| 102 | Tofacitinib  systemic | Letter  dermatology | UK  unicentric | 1/0/0/1  Research insterest | 0 | NR |
| 103 | Tofacitinib  systemic | Abstract  Congress  rheumatology | Multinational-  Argentima  Multicentric (7) | 10/0/4/6  Research insterest | NR | NR |
| 104 | Tofacitinib  systemic | Abstract  Congress  General  medicine | Switzerland  unicentric | 2/0/0/1  Research insterest | NR | NR |
| 105 | Itacitinib  systemic | Fulll text  dermatology | Multinational-EEUU  multicentric | 11/0/9/2  Pharma | 9 | Incyte Corporation |
| 106 | Tofacitinib  systemic | Fulll text  dermatology | EEUU  Unicentric | 4/0/4/0  Pharma | 4 | Pfizer |
| 107 | BMS 986165  systemic | Full text  General medicine | Multinational  EEUU  Multicentric (7) | 9/2/3/4  Research insterest | 9 | BristolMyers Squibb, |
| 108 | PF-06700841  systemic | Full text  Pharmacology | EEUU  Multicentric (2) | 11/0/10/1  Pharma | 11 | Pfizer |
| 109 | Tofacitinib  systemic | Abstract  Congress  dermatology | EEUU  Multicentric (7) | 9/0/6/3  Pharma | 9 | NR |
| 110 | Tofacitinib  systemic | Abstract  Congress  dermatology | multinational-EEUU  Multicentric (4) | 6/1/4/1  Pharma | NR | Pfizer |
| 111 | Tofacitinib  systemic | Abstract  Congress  dermatology | multinational-EEUU  Multicentic (4) | 4/0/2/2  Pharma-research | NR | Pfizer |
| 112 | Tofacitinib  systemic | Abstract  Congress  dermatology | EEUU  unicentric | 1/0/5/0  Pharma | NR | Pfizer |
| 113 | Tofacitinib  systemic | Abstract  Congress  dermatology | EEUU  unicentric | 1/0/5/0  Pharma | NR | Pfizer |
| 114 | Tofacitinib  systemic | Abstract  Congress  dermatology | multinational-EEUU  Multientric (3) | 4/0/2/3  Pharma-research | NR | Pfizer |
| 115 | Ruxolitinib  topical | Abstract  Congress  dermatology | EEUU  Multicentric (3) | 13/0/11/2  Pharma | NR | NR |
| 116 | Ruxolitinib  topical | Abstract  Congress  dermatology | EEUU  Multicentric (3) | 4/0/2/2  Pharma-research | NR | Incyte Corporation |
| 117 | Tofacitinib  systemic | Abstract  Congress  rheumatology | EEUU  Multicentric (3) | 10/0/8/2  Pharma | NR | NR |
| 118 | Brepocitinib  systemic | Full text  dermatology | EEUU  Multicentric | 0/0/8/3  Pharma | 11 | Pfizer |

ID: Reference identifier; Interest conflict: number of authors with conflicts interests; NR, not reported

**Table S8. Tofacitinib serious AE systemic.**

| **Clinical Trial ID/ Study reference** | **Arm group**  **time treatment** | **Tofacitinib**  **5 mg BID**  **Total/**  **Cardiac/**  **Infecction/**  **Cancer/** | **Tofacitinib**  **10 mg BID**  **Total/**  **Cardiac/**  **Infecction/**  **Cancer** | **Etanercept**  **50mg/weeks**  **Total/**  **Cardiac/**  **Infecction/**  **Cancer** | **Placebo**  **Total/**  **Cardiac/**  **Infecction/**  **Cancer** |
| --- | --- | --- | --- | --- | --- |
| NCT00678210 | 12 weeks | 1/1/0/0  n=49 | NA | NA | 0/0/0/0  n=50 |
| NCT01186074 | 24 weeks | 6/3/0/0  n=218 | 9/1/1/4  n=157 | NA | NA |
| NCT01241591 | 12 weeks | 7/1/2/0  n=329 | 5/0/2/1  n=330 | 7/0/2/0  n=335 | 2/0/0/0  n=107 |
| NCT01276639 | 52 weeks | 24/3/3/6  n=363 | 19/1/9/1  n=360 | NA | 2/0/0/0  n=45 |
| NTC01309737 | 52 weeks | 18/3/6/0  n=382 | 19/0/7/3  n=381 | NA | 2/1/0/0  n=44 |
| NCT01710046 | 16 weeks | NA | 0/0/0/0  n=9 | NA | 1/0/0/0  n=3 |
| NCT01519089 | 52 weeks | 3/3/0/0  n=47 | 3/0/1/0  n=47 | NA | NA |
| NCT01163253 | 66 months | NA | 304/39/88/65  n=2281 | NA | NA |
| NCT01815424 | 52 weeks | 4/1/1/0  n=88 | 2/0/0/1  n=90 | NA | 0/0/0/0  n=88 |
| NCT01736696 | 14 days | 0/0/0/0  n=5 | 1/0/1/0  n=5 | NA | 2/0/0/0  n=13 |
| Serum Human Beta-Defensin-2  [21]** | 16 weeks | 0/0/0/0  n=5 | 0/0/0/0  n=7 | NA | 0/0/0/0  n=6 |

*BID: twice daily; ** Table S2. NA: not applicable

**Table S9. Topical Tofacitinib oinment treatment efficacy/safety**

| **Clinical Trial ID** | 0,02%  EPB*/  TPPS**/PGA  tofacitinib  ointment  1 or 2 twice daily  AE total  most frequent AE  AE serious | 0,2%  EPB*/  TPPS**/  PGA***  Tofacitinib  ointment  1 or 2 twice daily  AE total  most frequent AE  AE serious | 1%  EPB*/  TPPS**/  PGA***  Tofacitinib  ointment  1 or 2 twice daily  AE total  most  frequent AE  A serious | 2%  EPB*/  TPPS**/  PGA***  Tofacitinib  ointment  1 or 2 twice daily  AE total  most frequent AE  AE serious | 4%  EPB*/  TPPS**/  PGA***  Tofacitinib  ointment  1 or 2 twice  daily  AE total  most frequent AE  AE serious | 50µg/g  calcipotriol  EPB*/  TPPS**/  PGA***  AE total  most frequent AE  AE serious | Vehicle  EPB*/  TPPS**/  PGA***  AE total  most frequent AE  AE serious |
| --- | --- | --- | --- | --- | --- | --- | --- |
| NCT  02193815  (12 days  Indicates events were collected by non-systemaic assessment | NA  NA  NA | NA  NA  NA | NA  NA  NA | Baseline  364.1  (135.82)-117.8  (115.15)/NA/NA  n=15  No AE serious  Not specified | Baseline:  358.9 (132.84)  day 12  17.7  (91.10)  /NA/NA  n=15  No AE serious  Not specified | 135.5  (114.27)  /NA/NA  n=15  No AE serious  Not specified | Baseline  353.1(121.03  day 12  32.9  (96.72)  NA/NA  n=15  No AE serious  Not  specificied |
| NCT  01246583  (4  weeks) | NA | NA | NA | NA/^*****^-54.4%-  -24.2%/NA  n=48  Burning/  stinging  No AE serious | NA  NA | NA  NA | NA^/*****^-51.5%  -17.2%/  NA  n=23  Burning/  stinging  No AE serious |
| NCT  01831466  (12 weeks)  Indicates events were collected by non-systematic assessment | NA | NA | NA/  21,6%-12,9%  n=144  69/7  Upper Respiratory tract infection | NA/  21,1%-20%  n=141  63/0  Nasopharyngitis | NA | NA | NA/  17,6%-  16,9%  n=145  73/4  Nasopharyingitis |
| NCT  00678561  (4 weeks) | NA/-3.29 (-9.03, 2.44; P=0.46) /NA  7/0/not  Specified  N=23 | NA/-1.78% (-7.30, 3.75; p=0.68)/NA N=23  13/0/ not  Specified | NA | NA/-4.36% (-10.05, 1.34; P=0.33)/  NA  N=23  13/0/ not  Specified | NA | NA | NA/-6.94 (-15.25, 1.38; P=0.28) /NA  N=12  7/0/ not specified |

* Change From Baseline in Psoriatic Skin Thickness/Echo-Poor Band (EPB) for PF-06263276 4% Solution in Comparison to Corresponding Vehicle at Day 12 [Time Frame: Day 1 (Baseline), Day 12] Psoriatic skin thickness was measured using a 20 megahertz (MHz) high frequency sonograph. Serial A-scans were composed and presented on a monitor as a section of the skin.

** Percent change Target Plaque Severity Score (TPSS) [Time Frame: 4 Weeks; ***12 weeks; ****4weeks; ]*****. Negative values for differences between groups represent a favourable treatment effect; NA; not applicable.

******** Percentage of Participants Achieving a PGA-C Response of Clear (0) or Almost Clear (1) and Greater Than or Equal to (≥) 2 Grade / Point Improvement From Baseline at Week 12

**Table S10. Topical ruxolitinib ointment treatment efficacy/safety**

| **Clinical Trial ID** | Ruxolitinib phosphate cream 0.5%QD^*^  LAPCB^***^  AE total  /serious/  Most frequent  28 days | Ruxolitinib phosphate cream 1.0%QD^*^  LAPCB^***^  AE total  /serious/  Most frequent  28 days | Ruxolitinib phosphate cream 1.5%BID^**^  LAPCB^***^  AE total  /serious/  Most frequent  28 days | Calcipotriene 0.005%  LAPCB^***^  AE total  /serious/  Most frequent  28 days | Betamethasone dipropionate 0.05% cream  LAPCB^***^  AE total  /serious/  Most frequent  28 days | Placebo  LAPCB^***^  AE total  /serious/  Most frequent  28 days |
| --- | --- | --- | --- | --- | --- | --- |
| NCT00820950 | n=5  2.30%(14,18%)  NA/0/NA | n=6  -9.92% (10.31%)  NA/0/NA | n=15  -20.68%  (9.71%)  NA/0/NA | n=5  -1.28% (15.87%)  NA/0/NA | n=5  -3.56%  (17.58%)  NA/0/NA | n=17  -4.67%  (6.89%)  NA/0/NA |
| NCT00617994 | NA | NA | NA | NA  NA  NA | NA  NA  NA | NA  NA  NA |
| NCT00778700  *** | NA | NA | NA | NA  NA | NA  NA | NA |

*QD: once daily; **BID: twice daily; ***Lesion area (percent change from baseline [SEM]) for various treatments: LAPCB

****Absolute change from Baseline to Day 84 in total lesion score for all treatable psoriatic lesions. [Time Frame: Baseline and Day 84 (or early study termination visit)]. Sum of erythema, scaling, and thickness for all treatable lesions; NA, not applicable

**Table S11. Peficitinib treatment efficacy/safety**

|  | ASP015K  10mg BID^**^  PASI 75  AE total/AE  Serious/ Most  frequent  6weeks | ASP015K  25mg  BID^**^  PASI 75  AE total/AE  Serious/ Most  frequent  6weeks | ASP015K  60 mg  BID^**^  PASI 75  AE total/AE  Serious/ Most  frequent  12 weeks | ASP015K  100mg  BID^**^  PASI 75  AE total/AE  Serious/ Most  frequent  6weeks | ASP015K  50mg  QD^*^  PASI 75  AE total/AE  Serious/ Most  frequent  6weeks | Placebo  PASI 75  AE total/AE  Serious/ Most  frequent  6 weeks |
| --- | --- | --- | --- | --- | --- | --- |
| NCT01096862 | n=19  31,6%  11/ NA /  Nasal congestion | n=21  14,3%  9/NA/  Dry mouth | n=19  57,9%  7/NA/  Nasopharyngitis | n=17  64,7%  11/NA/  Nasopharyngitis | n=19  15,8%  6/NA/  Headache | n=29  3,4%  11/NA/  Nasopharyngitis |

*QD: once daily; *BID: twice daily; NA, not applicable

**Table S12. Baricitinib treatment efficacy/safety**

|  | 2mgQD^*^  PASI75/  AE total  /serious/  Most frequent  12 weeks | 4mg QD^*^  PASI 75  AE total  /serious/  Most frequent  12 weeks | 8mgQD^*^  PASI75  AE total  /serious/  Most frequent  12 weeks | 10mg QD^*^  PASI75  AE total  /serious/  Most frequent  12 weeks | Placebo  PASI75  AE total  /serious/  Most frequent  12 weeks |
| --- | --- | --- | --- | --- | --- |
| NCT01490632 | n=28  28,6%  9/1/  Fatigue^**^ | n=63  28,6%  12/1/  Urinary  tract  infection^**^ | n=56  42,9%  21/1/  Nasopharyngitis^**^ | n=61  54,1%  23/1/  Nasopharyngitis^**^ | N=30  16,7%  11/1/  Nasopharyngitis^**^ |

*QD: once daily ; **Indicates events were collected by systematic assessment

**1**

**Table S13. Baricitinib serious AE**

|  | Arms group  treatments | Baricitinib  2mg | Baricitinib  4 mg | Baricitinib  8 mg | Baricitinb  10 mg | Placebo |
| --- | --- | --- | --- | --- | --- | --- |
| NCT01490632  (yes) | 24 weeks | 1  n=32  psoriasis* | 1  n=72  oesophageal carcinoma* | 1  n=64  squamous cell carcinoma of skin* | 1  n=69  pneumonia* | 1  n=34  diabetic  food* |

*Indicates events were collected by systematic assessment

**Table S14. Solcitinib treatment efficacy/safety**

|  | Solcitinib 100 mg PASI 75  AE total/AE  Serious/ Most  frequent  12 weeks | Solcitinib 200mg PASI 75  AE total/AE  Serious/ Most  frequent  12 weeks | Solcitinib 400 mg PASI 75  AE total/AE  Serious/ Most  frequent  12 weeks | Placebo  PASI 75  AEtotal/AE  Serious/ Most  frequent  12 weeks |
| --- | --- | --- | --- | --- |
| NCT01782664  (yes) | n=15  13%  10/2/headache* | n=16  25%  14/0/ Nasopharyngitis^*^ | n=14  57%  10/1/Nasopharyngitis^*^ | n=14  0%  13/0/Headache* |

*Indicates events were collected by systematic assessment. AE:adverse events, PASI 75: 75% reduction psoriasis area severity index

**Table S15. Solcitinib serious AE**

|  | Armn groups | Solcitinib  100 mg BID* | Solcitinib  200 mg  BID* | Solcitinib  400 mg  BID* | Placebo |
| --- | --- | --- | --- | --- | --- |
| NCT01782664 | 12 weeks | 3  n=15  trombocitopenia  concussion  ligament ruptura** | 0  n=16 | 1  n=14  abdominal pain** | 0  n=14 |

**BID: twice daily,**Indicates events were collected by systematic assessment

**Table S16. Itacitinib treatment efficacy/safety**

|  | Itacitinib 100 mg QD*  PGAs change/  AE total/  Most frequent AE/ | Itacitinib  200 mg QD*  PGAschange  AE total/  Most frequent AE/ | Itacitinib  200 mgBID**  PGAschange  AE total/  Most frequent AE/ | Itacitinib  600 mg QD*  PGAschange  AE total/  Most frequent AE/ | Placebo  PGAs change  AEtotal/  Most frequent AE/ |
| --- | --- | --- | --- | --- | --- |
| NCT01634087 | -22.2%/3/  Nasopha  ryngitis  Headache  n=9 | -29,4%/5/  Nasopha  ryngitis  AST increased  n=9 | -35,2%/6/  Nasopha  Ryngitis  n=9 | -42,4%/5/  Nasopha  Ryngitis  N=11 | -12,5%/4  Nasopha  Ryngitis  N=12 |

*QD: once daily ,*BID: twice daily, AE:advere events

**Table S17. Itacitinib serious AE**

|  | Itacitinib 100 mg QD | Itacitinib  200 mg QD | Itacitinib  200 mgBID | Itacitinib  600 mg QD | Placebo |
| --- | --- | --- | --- | --- | --- |
| NCT01634087 | 0 | 0 | 0 | 0 | 0 |

*QD: once daily ,*BID: twice daily

**Table S18.-Deucravacitanib/ BMS-986165 treatment efficacy/safety**

|  | BMS-986165 6mgBID*  PASI 75/  AE total/AE  Serious/ Most  frequent  4 weeks | BMS-986165 QD PASI 75  AE total/AE  Serious/ Most  frequent  4 weeks | Placebo  PASI 75  AE total/AE  Serious/ Most  frequent  4 weeks |
| --- | --- | --- | --- |
| NCT02931838 | n=44  75%  25/0/Nasopharyngithis ^*^ | n=45  66,4%  19/0/ Blood creatinine  increased^*^ | n=45  6,7%  13/1/0/Nasopharyngitis^*^ |
| NCT03924427 | NA  NA  NA | NA  NA  NA | NA  NA  NA |
| NCT04772079 | NA  NA  NA | NA  NA  NA | NA  NA  NA |
| NCT04036435- | NA  NA  NA | NA  NA  NA | NA  NA  NA |
| NCT03624127 | NA  NA  NA | NA  NA  NA | NA  NA  NA |
| NCT04167462 | NA  NA  NA | NA  NA  NA | NA  NA  NA |
| NCT03611751- | NA  NA  NA | NA  NA  NA | NA  NA  NA |
| NCT02534636 | NA  NA  NA | NA  NA  NA | NA  NA  NA |

*QD: once daily *BID: twice daily,*Indicate events were collected by systematic assesstment, AE:adverse events;

PASI 75:75% reduction psoriasis area severity index. NA, not applicable

**Table S19.- Abrocitinib/PF-04965842 treatment efficacy/safety**

|  | PF-04965842  200mg QD^*^  reduction PASI  AEtotal/AE  Serious/ Most  frequent  4weeks | PF-04965842  400mg QD^*^  reduction PASI  AEtotal/AE  Serious/ Most  frequent  4weeks | PF-04965842  200mg BID^**^  reduction PASI  AE total/AE  Serious/ Most  frequent  4weeks | Placebo  Reduction PASI  AE total/AE  Serious/ Most  frequent  4weeks |
| --- | --- | --- | --- | --- |
| NCT02201524 | N=15  -11,73%  7/0/  Nausea^***^ | N=16  -13,09%  14/0/  Headache^***^ | N=14  -13,71%  11/0/  Trombocitopenia^***^ | N=14  -6,61%  6/1/  Headache^***^ |

*QD: once daily, **BID: twice daily,***Indicates events were collected by non-systematic assessment

PASI: reduction psoriasis area severity index; AE: adverse event

**Table S20. Brepocitinib/ PF-06700841 treatment efficacy/safety**

|  | PF-06700841 30mgQD*  reduction PASI  AE total/AE  Serious/ Most  frequent  4weeks | PF-06700841 100mgQD*  reduction PASI  AE total/AE  Serious/ Most  frequent  4weeks | Placebo  reduction PASI  AE total/AE  Serious/ Most  frequent  4weeks |
| --- | --- | --- | --- |
| NCT02310750 | n=14  NA  11/0/Blood creatinine  increased** | n=9  NA  6/0/ Blood creatinine  increased** | n=7  NA  5/0/constipation** |
|  | PF-06700841 30mgQD*  reduction PASI 75  AE total/AE  Serious/ Most  frequent  12weeks | PF-06700841 30mgQD* 4 weeks  Induction followed 100 mg once weekely PASI  AE total/AE  Serious/ Most  frequent  12weeks | Placebo  reduction PASI  AE total/AE  Serious/ Most  frequent  12weeks |
| NCT02969018 | n=_25  86%  13/0/nasopharyngitis | n=19  36.7%  16/0/nasopharyngitis | N=17  13%  9/0/nasopharyngitis |
| NCT03850483 | NA  NA  NA | NA  NA  NA | NA  NA  NA |

*QD: once daily; **Events were collected by non-systematic assessment; AE:adverse events, PASI:Psoriasis Area Severity Index; NA; not applicable
